# Strain-dependent *toxT* expression, rather than ToxT activity, governs virulence gene regulation in *Vibrio cholerae*

**DOI:** 10.3389/fmicb.2026.1755947

**Published:** 2026-02-19

**Authors:** Jayun Joo, Donghyun Lee, Seoyun Choi, Hyungoo Kim, Dong Wook Kim, Eun Jin Kim

**Affiliations:** 1 Department of Pharmacy, College of Pharmacy, Hanyang University, Ansan, Republic of Korea; 2 Institute of Pharmacological Research, Hanyang University, Ansan, Republic of Korea

**Keywords:** cholera, cholera toxin, TCP major subunit A, toxin coregulated pilus, ToxT, *Vibrio cholerae*

## Abstract

The AraC-type transcriptional activator ToxT is a central regulator of *Vibrio cholerae* virulence, directly controlling expression of the major virulence genes *ctxAB* and *tcpA*. Although biotype-specific culture conditions have been widely used to study virulence gene regulation, virulence gene expression patterns are not conserved across *V. cholerae* strains, particularly within the El Tor biotype. In this study, we investigated strain- and allele-dependent control of *toxT* expression using isogenic derivatives harboring chromosomally encoded His-tagged *toxT* alleles, enabling direct comparison of *toxT* transcription, ToxT protein production, and downstream virulence gene activation. All *toxT* alleles retained transcriptional activator function once expressed; in the classical biotype strain O395, four different *toxT* alleles activated virulence gene expression, although the expression levels of *tcpA* and *ctxAB* varied among alleles. In contrast, in the El Tor prototype strain N16961, *toxT*-AY and *toxT*-AF supported virulence gene expression at 30°C, whereas *toxT*-SY and *toxT*-SF were transcriptionally repressed. By comparison, the atypical El Tor strain IB5230 exhibited robust expression of *toxT*-SY and *toxT*-SF, with activation of downstream virulence genes even at 37°C, a temperature relevant to the intestinal environment. Together, these results demonstrate that *toxT* expression functions as a regulatory gate within the canonical ToxR regulon and represents a critical control point governing strain-specific virulence gene regulation and pathogenic potential in *V. cholerae*.

## Introduction

*Vibrio cholerae* is a Gram-negative bacterium and the etiological agent of cholera, a severe diarrheal disease that remains a major public health challenge, particularly in regions with inadequate sanitation and water supply systems (Sack et al., 2004; Clemens et al., 2017). Based on the structure of its O-antigen, *V. cholerae* is classified into more than 200 distinct serogroups; however, strains belonging to the O1 and O139 serogroups are primarily responsible for epidemic cholera (Sack et al., 2004; Clemens et al., 2017; Mutreja et al., 2011). The clinically significant O1 serogroup is further subdivided into three serotypes: Ogawa, Inaba, and Hikojima (Sack et al., 2004).

Additionally, O1 serogroup strains are classified into two biotypes, classical and El Tor, based on distinct microbiological characteristics, including biochemical properties, phage susceptibility, and environmental resilience (Clemens et al., 2017). Historically, the classical biotype was responsible for the first six cholera pandemics recognized since the early 19th century. In contrast, the ongoing seventh cholera pandemic, which began in 1961, represents a notable shift in disease epidemiology, as it has been predominantly attributed to El Tor biotype strains (Mutreja et al., 2011; Kim et al., 2014; Kim et al., 2015).

The ability of *V. cholerae* to cause disease is primarily driven by two key virulence factors: cholera toxin (CT) and toxin co-regulated pilus (TCP). CT, a potent enterotoxin secreted by strains within the epidemic serogroups O1 and O139, induces the profuse watery diarrhea characteristic of cholera, leading to severe dehydration and, if untreated, potentially fatal outcomes (Kanungo et al., 2022). TCP plays a crucial role in the initial stages of infection by facilitating the colonization of the human small intestine, which is essential for the pathogen’s persistence and proliferation within the host.

*V. cholerae* relies on a complex regulatory network, the ToxR regulon, to ensure proper activation of CT and TCP in response to host environmental cues (Herrington et al., 1988; Ghosh et al., 2014; Goss et al., 2013; Canals et al., 2023; Childers and Klose, 2007). Central to this regulon is ToxT, an AraC-type transcriptional activator that directly regulates the expression of the virulence genes *ctxAB* and *tcpA*, which encode CT and the major structural component of TCP, TcpA, respectively (Cruite et al., 2019).

ToxT functions by binding to specific DNA sequences within the promoter regions of these virulence genes, initiating their transcription. Experimental studies, including electrophoretic mobility shift assays (EMSA), reporter gene assays, and three-dimensional structural analyses, have demonstrated that ToxT directly activates the transcription of these target genes, leading to the subsequent production of CT and TCP (Abuaita and Withey, 2011; Lowden et al., 2010; Hulbert and Taylor, 2002; Thomson and Withey, 2014).

Despite extensive research on virulence gene regulation, significant variability exists in virulence gene expression among different *V. cholerae* strains under laboratory conditions (Jonson et al., 1990). Classical biotype strains typically express virulence genes when cultured in aerated LB medium at pH 6.5 and 30°C (agglutinating conditions) (Waldor and Mekalanos, 1996), whereas El Tor biotype strains require the specialized AKI culture method, a two-phase process involving static incubation in AKI medium (composed of 1.5% Bacto peptone, 0.4% yeast extract, 0.5% NaCl, and 0.3% NaHCO_3_) at 37°C, followed by vigorous shaking (Sanchez et al., 2004). However, some strains from both biotypes fail to express virulence genes even under their respective inducing conditions, suggesting the involvement of additional regulatory mechanisms (Lee et al., 2023; Baek et al., 2020).

In most *V. cholerae* strains, including both classical and El Tor biotypes, the ToxT protein shares the same amino acid sequence and carries the *toxT*-SY allele, with Ser at position 65 and Tyr at position 139 (Cruite et al., 2019). However, recent studies have identified variant *toxT* alleles with distinct amino acid substitutions (Lee et al., 2023; Kim et al., 2022). The *toxT*-AY allele, found in the classical biotype strain 569B, is characterized by Ala at position 65 and Tyr at position 139. Meanwhile, the *toxT*-SF allele, present in the El Tor biotype strain MG116025, contains Ser at position 65 and Phe at position 139 (Kim et al., 2017). Additionally, an artificial allele, *toxT*-AF, has been generated by combining the *toxT*-AY and *toxT*-SF alleles, resulting in Ala at position 65 and Phe at position 139 (Lee et al., 2023).

Since the *toxT*-SF allele has been shown to facilitate virulence gene expression in several *V. cholerae* strains, previous studies have investigated the effects of replacing the authentic *toxT* allele with alternative alleles in a variety of *V. cholerae* backgrounds (Lee et al., 2023; Kim et al., 2022; Kim et al., 2017). When introduced into various *V. cholerae* strains, these *toxT* alleles exhibited distinct effects on CT and TCP expression under laboratory culture conditions (Lee et al., 2023; Kim et al., 2017). In the classical biotype strain O395, all four alleles activated expression of virulence genes when cultured in LB medium at 30°C (Lee et al., 2023). In contrast, El Tor biotype strains generally do not express virulence genes under these conditions. Specifically, in the El Tor prototype strain N16961, *toxT*-SY and *toxT*-SF failed to induce virulence gene expression in LB medium, whereas *toxT*-AY and *toxT*-AF promoted expression in aerated LB cultures (Lee et al., 2023; Choi et al., 2023). Notably, in the atypical El Tor strain IB5230, which was responsible for the 2010 Haitian cholera outbreak, the regulatory pattern was completely reversed: *toxT*-SY and *toxT*-SF promoted virulence gene expression, while *toxT*-AY and *toxT*-AF did not when cultured in LB medium (Kim et al., 2022; Choi et al., 2023; Lee et al., 2024).

Variation in virulence gene expression across *V. cholerae* strains and *toxT* alleles reflects the complexity of regulation, suggesting that genetic background, regulatory circuitry, and environmental responsiveness together shape pathogenic potential.

This study aimed to clarify whether differences in virulence gene expression among *V. cholerae* strains are attributable to the expression of specific *toxT* alleles. In particular, we sought to evaluate whether certain *toxT* alleles are transcriptionally silent or, alternatively, are expressed but fail to activate downstream virulence genes in strain–allele combinations that do not support virulence gene expression. To address this, we conducted a comprehensive analysis of *toxT*, *ctxAB*, and *tcpA* transcript levels using qRT-PCR, and validated these findings at the protein level by Western blotting across multiple *V. cholerae* strains.

Significant differences in *toxT* expression—and consequently, in the activation of CT and TCP production—were observed depending on the *toxT* allele and strain. Our results suggest that the regulation of virulence gene expression in *V. cholerae* is more complex and strain-dependent than previously understood.

## Materials and methods

### Bacterial strains

*V. cholerae* strains and their isogenic derivatives are listed in Supplementary Table S1. A *V. cholerae* clinical isolate from the 2010 Haiti cholera outbreak, IB5230, and the *V. cholerae* type strains O395, 569B, and N16961 were obtained from the culture collections of the International Vaccine Institute, Seoul, Korea (Mutreja et al., 2011; Kim et al., 2014). Isogenic derivatives of these *V. cholerae* strains carrying different *toxT* alleles were part of our laboratory collection or were newly constructed in this study (Lee et al., 2023; Baek et al., 2020; Kim et al., 2022).

### *toxT* allele exchange

Allele exchange at the 139th amino acid position: Allelic replacement of the chromosomal *toxT* gene with alternative *toxT* alleles in *V. cholerae* strains was conducted using established allelic-exchange procedures (Kim et al., 2014; Lee et al., 2023; Kim et al., 2017). Primers used for constructing isogenic derivatives of *V. cholerae* strains are listed in Supplementary Table S2. An 843-bp region of *toxT*—from 50 nucleotides upstream of the start codon to nucleotide 793—was amplified by PCR with primers toxT-XbaIF and toxT-SacIR using genomic DNA from MG116025 (*toxT-SF*) and N16961 (*toxT-SY*) as templates. Each PCR product was cloned into the suicide plasmid pCVD442 (Miller and Mekalanos, 1988), yielding pCVD-toxT-SF and pCVD-toxT-SY. A single-nucleotide polymorphism (SNP) at position 416—A416 in N16961 and T416 in MG116025—is located centrally within these fragments. These constructs were introduced into the respective *V. cholerae* strains, and allelic replacement was performed using standard pCVD442-mediated allelic-exchange procedures to swap *toxT*-SF and *toxT*-SY alleles between strains. Through this procedure, *toxT*-SF in MG116025 was replaced with *toxT*-SY, and the native *toxT*-SY alleles in O395 and N16961 were substituted with *toxT*-SF (Kim et al., 2014; Kim et al., 2017).

The classical biotype strain 569B contains a distinct *toxT* allele (*toxT*-AY). Conjugal delivery of pCVD-toxT-SF into 569B was used to introduce the *toxT*-AF allele, following the same pCVD442-mediated allelic-exchange procedure described above. Allele replacement and resolution of the pCVD442 vector were verified by Sanger sequencing.

Allele exchange at the 65th amino acid position: A series of allele-exchange plasmids was used to generate isogenic *V. cholerae* derivatives carrying SNPs at amino acid positions 65 and 139 of *toxT*. Four 1,308-bp DNA fragments—corresponding to the region from nucleotide 709 of *tcpF* to nucleotide 793 of *toxT*—were amplified by PCR using the primer pair TcpF-XbaIF and toxT-SacIR. Templates for amplification included genomic DNA from strains N16961, YJB003 (N16961-*toxT*-SF), 569B, and EJK007 (569B-*toxT*-AF) (Baek et al., 2020).

The PCR products were subcloned into the suicide vector pCVD442 to generate pCVD-toxT-Out-SY, pCVD-toxT-Out-SF, pCVD-toxT-Out-AY, and pCVD-toxT-Out-AF. Each construct was then introduced into the corresponding *V. cholerae* strains by conjugation, and allelic exchange was carried out to replace the native *toxT* allele with the corresponding alternative allele. Recombinants that had resolved the pCVD442 vector were selected on sucrose-containing agar plates, and the intended *toxT* alleles were confirmed by Sanger sequencing.

### Construction of a *toxT-*deleted (Δ*toxT*) isogenic variant of *V. cholerae* strains

A 635-bp DNA fragment spanning from nucleotide 709 of *tcpF* to nucleotide 120 of *toxT* was amplified by PCR using the primers TcpF-KpnIF and TcpF-BamHIR (Lee et al., 2023). This fragment amplified from the genomic DNA of *V. cholerae* N16961 was inserted into the KpnI and BamHI sites of pUC18 to generate pUC-tcpF. A second 660-bp fragment, spanning nucleotide 616 of *toxT* to nucleotide 435 of *tcpJ*, was amplified using primers TcpJ-BamHIF and TcpJ-PstIR and cloned into the BamHI and PstI sites of pUC-tcpF, producing pUC-tcpF-tcpJ. Using primers TcpF-XbaIF and TcpH-SacIR, a 1,301-bp fragment was then amplified from pUC-tcpF-tcpJ and subcloned into the suicide vector pCVD442 to construct pCVD-del-toxT. This construct was conjugally transferred into *V. cholerae* strains to obtain *toxT*-deleted (Δ*toxT*) isogenic derivatives through allelic exchange. After resolution of the pCVD442 vector, deletion of the targeted internal *toxT* region was verified by Sanger sequencing. The resulting Δ*toxT* isogenic derivatives lacked 165 internal amino acids of ToxT corresponding to residues 41–205.

### Construction of pBAD-toxT-His recombinant plasmids

Four 852-bp DNA fragments were PCR-amplified, each containing the 828-bp *toxT* open reading frame (ORF) lacking the stop codon, a GCC codon inserted between amino acids 1 and 2 to maintain the reading frame, an 18-bp sequence encoding a C-terminal (His)_6_-tag, and a TAA termination codon. PCR amplification was performed using primers toxT-NcoIF and toxT-SalIHisR with genomic DNA from *V. cholerae* O395 (*toxT*-SY) and its allele-exchange derivatives YJB001 (*toxT*-SF), EJK008 (*toxT*-AY), and EJK009 (*toxT*-AF). Each PCR product was digested with NcoI and SalI and ligated into the corresponding sites of the expression vector pBAD24 (Guzman et al., 1995), generating the constructs pBAD-toxT-SY-His, pBAD-toxT-SF-His, pBAD-toxT-AY-His, and pBAD-toxT-AF-His.

The resulting plasmids were transformed into *E. coli* DH5α and *V. cholerae* DHL010 (N16961-Δ*toxT*). Expression of His-tagged ToxT from the P_BAD_ promoter was induced by supplementing cultures with L-arabinose to the bacterial culture at a final concentration of 0.2%. Production of the His-tagged ToxT protein was confirmed by Western blot analysis using an anti-His-tag (Cell Signaling, Danvers, MA, USA).

### Construction of isogenic variants of *V. cholerae* strains that contain *toxT*-His

The *tcpJ* sequence is identical in both classical and El Tor *V. cholerae* O1 strains. A 762-bp *tcpJ* fragment was PCR-amplified from O395 genomic DNA using primers TcpJ-SalIF and TcpJ-SalIR. The PCR product was digested with SalI and ligated into the SalI site of the plasmids pBAD-toxT-SY-His, pBAD-toxT-SF-His, pBAD-toxT-AY-His, and pBAD-toxT-AF-His, generating pBAD-toxT-SY-His-tcpJ, pBAD-toxT-SF-His-tcpJ, pBAD-toxT-AY-His-tcpJ, and pBAD-toxT-AF-His-tcpJ, respectively. The orientation of the inserted *tcpJ* fragment was confirmed by DNA sequencing (Supplementary Figure S1).

Using the recombinant plasmids as templates, four 1,620-bp DNA fragments spanning from the initiation codon of His-tagged *toxT* to the termination codon of *tcpJ* were PCR-amplified with primers pBAD-toxT-XbaIF and pBAD-tcpJ-SacIR. The amplified products were digested with XbaI and SacI and inserted into the corresponding sites of the suicide vector pCVD442, generating pCVD442-toxT-SY-His-tcpJ, pCVD442-toxT-SF-His-tcpJ, pCVD442-toxT-AY-His-tcpJ, and pCVD442-toxT-AF-His-tcpJ.

These recombinant plasmids were conjugally transferred into the corresponding isogenic *toxT* allele derivatives of the O395, N16961, and IB5230 strains to construct isogenic derivatives harboring His-tagged *toxT* through the allelic exchange method. Excision of the suicide vector and replacement of *toxT* with *toxT*-His were screened on sucrose-containing agar plates, and the DNA sequence of the His-tagged *toxT* was confirmed by Sanger sequencing (Supplementary Figure S1).

### CTX phage transduction

An isogenic derivative of *V. cholerae* N16961, referred to as PM20, in which the *ctxAB* genes had been replaced by a kanamycin resistance cassette, served as the CTXΦ donor strain (Kim et al., 2017). PM20 was grown in LB medium supplemented with 20 ng/mL mitomycin C to induce CTXΦ production (Waldor and Mekalanos, 1996). Following induction, the culture was centrifuged, and the clarified supernatant containing CTXΦ particles was collected. This phage-containing supernatant was then mixed with recipient O395 cells that had been prepared under agglutinating conditions (30°C in LB medium, pH 6.5). After a 30-min incubation to allow CTXΦ infection, the mixture was plated onto LB agar plates containing kanamycin (100 μg/mL) to select for O395 transductants that had acquired CTXΦ.

Kanamycin-resistant colonies were isolated, and the presence of the replicative form of the phage genome, pCTX-1kan, was verified by Sanger sequencing of the intergenic region between the kanamycin resistance and *rstR*. CTX-1kan virions produced from *V. cholerae* O395:pCTX-1kan cultures were subsequently used to transduce the recipient strains O395 (*toxT*-SY), O395-H (*toxT-*SY-His), EJK009 (*toxT*-AF), and EJK009-H (*toxT-*AF-His). Recipient cells were prepared under agglutinating conditions or cultured in LB medium at 37°C. Transduction efficiency was calculated as the ratio of kanamycin-resistant transductants to the total number of recipient cells.

### SDS-PAGE and Western blotting

Approximately 3 × 10^8^ cells (for His-tagged ToxT) or 1 × 10^7^ cells (for TcpA) were subjected to SDS-PAGE using 12 and 15% polyacrylamide gels, respectively. The PageRuler Prestained Protein Ladder (Thermo Scientific, Cat. No. 26616) was used as a molecular weight marker to estimate the apparent sizes of His-tagged ToxT and TcpA proteins. Following electrophoresis, proteins were transferred onto nitrocellulose membranes. Western blot analysis was then performed using anti-His antibody (Cell Signaling Technology, Danvers, MA, USA) and anti-TcpA antibody [a generous gift from Dr. W. F. Wade, Dartmouth University, Hanover, NH, USA (Taylor et al., 2004)]. Horseradish peroxidase (HRP)-conjugated goat anti-rabbit IgG (Cell Signaling Technology, 7074S) was used as the secondary antibody.

### RT-PCR and statistical analysis

A real-time qRT-PCR assay was performed as previously described, with some modifications (Abuaita and Withey, 2011). Total RNA was extracted from approximately 2 × 10^9^ cells at each time point using the Nextractor-48 N® nucleic acid extractor system (Genolution, Seoul, Korea), followed by treatment with RNase-free DNase 1. cDNA was synthesized with 1 μg of RNA using the Thermo Scientific RevertAid First Strand cDNA synthesis kit (ThermoFisher). The concentrations of RNA and cDNA were determined using NanoDrop 2000 (ThermoFisher). Real-time PCR was carried out using 100 ng of synthesized cDNA and specific primers (Supplementary Table S2) with a CFX96RT-PCR system and iQ™ SYBR® Green Supermix (BioRad). The PCR conditions were 95°C for 2 min, followed by 35 cycles of 95°C for 10 s, 60°C for 30 s, and 72°C for 30 s. Each run concluded with a melting-curve ranging from 55°C to 95°C to validate specific amplification. Expression values were normalized to the housekeeping gene *gyrA* as described previously (Abuaita and Withey, 2011; Ray et al., 2011; Raskin et al., 2020). The relative expression levels of *toxT*, *tcpA*, and *ctxAB* were calculated using the 2^−ΔΔCT^ method (Raskin et al., 2020). The expression levels of *toxT*, *tcpA*, and *ctxAB* at each time point were individually normalized to the expression levels of the corresponding genes in the 4-h culture of O395-H (O395-*toxT*-SY-His). All quantitative data are presented as the mean ± standard deviation (SD) from three independent biological replicates.

For statistical evaluation, each condition was compared with this reference condition. At each time point, statistical significance was assessed using one-way analysis of variance (ANOVA) followed by Dunnett’s multiple-comparison test, with the 4-h O395-H condition used as the control group. Only conditions that showed statistically significant decreases relative to the reference condition are indicated with asterisks in the figures; conditions that were not significantly different from or higher than the reference are not annotated. All statistical analyses were performed using GraphPad Prism software (version 9.2.0) and SPSS Statistics (version 29.0). A *p* value of < 0.05 was considered statistically significant.

## Results

### Functional characterization of *toxT* alleles

All four native *toxT* alleles exhibited transcriptional activator functions in the classical biotype strain O395 (Lee et al., 2023). However, only certain alleles (e.g., *toxT*-AY and *toxT*-AF in N16961, and *toxT*-SY and *toxT*-SF in IB5230) detectable activity in other strain backgrounds (Lee et al., 2023). To confirm that all four *toxT* alleles maintain their transcriptional activator functions in El Tor biotype strains, we analyzed virulence gene expression by exogenously expressing *toxT* alleles in a *toxT*-deleted N16961 strain.

An isogenic derivative of the N16961 strain, DHL010 (N16961-Δ*toxT*) was individually transformed with recombinant pBAD plasmids, each carrying a different His-tagged *toxT* allele (Lee et al., 2023). The expression of His-tagged ToxT and TcpA was assessed in the presence of 0.2% arabinose or 0.5% glucose. The addition of glucose effectively suppressed pBAD-driven basal expression of His-tagged ToxT. Since the O395 and IB5230 strains lose viability in the presence of 0.5% glucose due to impaired neutral fermentation, this experiment was performed exclusively in N16961 (Lee et al., 2023). All four *toxT* alleles were successfully induced by the P_BAD_ promoter, leading to the expression of both His-tagged ToxT and TcpA in the presence of 0.2% arabinose. In contrast, neither ToxT nor TcpA was detected in the presence of 0.5% glucose (Supplementary Figure S2).

These results indicate that, once expressed at comparable levels in N16961, the *toxT* alleles exhibit no significant functional differences. This observation aligns with previous studies showing that all four *toxT* alleles in the O395 strain can activate virulence gene expression (Lee et al., 2023; Lee et al., 2024).

### Functional equivalence of His-tagged ToxT compared to native ToxT

The introduction of a His-tag at the C-terminus of *toxT* alters the amino acid sequence of both ToxT and the adjacent TcpJ protein (Supplementary Figure S1), requiring an evaluation of the functional integrity of His-tagged ToxT (ToxT-His) relative to native ToxT. To assess the potential impact of these modifications, two complementary approaches were employed. First, the growth dynamics of isogenic derivatives expressing ToxT-His were compared to those of the parental strain expressing authentic ToxT under identical culture conditions. Second, the assembly and functionality of the TCP pilus—regulated by ToxT—were evaluated by measuring CTX phage transduction efficiency. Together, these analyses provide a comprehensive assessment of the functional equivalence of ToxT-His and native ToxT, enabling an evaluation of the effects of expression of His-tagging on ToxT and its downstream regulatory roles.

### Comparison of growth curves between isogenic derivatives harboring native *toxT* and His-tagged *toxT*

To assess whether integration of a His-tag at the C-terminus of the chromosomal *toxT* gene affects bacterial growth, growth curves were compared across four isogenic derivatives of each *V. cholerae* strain (Supplementary Figure S3). These included the parental strain containing the authentic *toxT* allele (*toxT*-SY), its derivative harboring the His-tagged *toxT* (*toxT*-SY-His), an isogenic derivative with an alternative *toxT* allele (*toxT*-AF), and its counterpart with the His-tagged alternative *toxT* allele (*toxT*-AF-His).

Bacteria were cultured in LB medium at pH 6.5 and 30°C, and their growth was monitored. After a brief lag phase, exponential growth was observed for up to 6 h of cultivation, with cell densities reaching approximately 5 × 10^9^ CFU/mL at the stationary phase. The growth curves of the isogenic derivatives of each strain were nearly identical, indicating that replacing the authentic *toxT* with either a His-tagged *toxT* or an alternative *toxT* (including His-tagged alternative *toxT*) did not significantly affect the growth rate (Supplementary Figure S3).

### Production of functional TCP by alternative *toxT* alleles

The functional assembly of the TCP pilus, mediated by TcpA expressed under the control of His-tagged ToxT, was evaluated by measuring CTX*Φ* transduction efficiency (Kim et al., 2014; Kim et al., 2017). Transduction experiments were conducted using the CTX Φ-1Km (in which *ctxAB* was replaced by a kanamycin-resistance cassette), produced from the replicative form of the CTX phage. Four isogenic derivatives of the *V. cholerae* strain O395 were used as recipients: O395 (*toxT*-SY), O395-H (His-tagged *toxT*-SY), EJK009 (*toxT*-AF), and EJK009-H (His-tagged *toxT*-AF). Transduction efficiency was determined as the ratio of transductants to the total number of recipient cells (Waldor and Mekalanos, 1996; Kim et al., 2017; Yu et al., 2018).

The observed CTXΦ transduction efficiencies were approximately 12.9% for O395, 12.8% for O395-H, 26.6% for EJK009, and 27.0% for EJK009-H (Supplementary Table S3). Consistent with previous reports that the *toxT*-AF allele enhances *tcpA* expression relative to *toxT*-SY, EJK009 (*toxT*-AF) and EJK009-H exhibited higher transduction efficiencies compared to O395 (*toxT*-SY) and O395-H (Lee et al., 2023; Kim et al., 2022). In contrast, when recipient cells were prepared in LB medium at 37°C, transduction efficiency was reduced by more than 10^7^-fold, indicating that functional TCP pili were not assembled under these conditions (Waldor and Mekalanos, 1996).

These results suggest that functional TCP was assembled, with no substantial differences in transduction efficiency between isogenic derivatives expressing His-tagged ToxT and those expressing native ToxT. Moreover, the findings indicate that the addition of the His-tag did not noticeably affect TcpA production, TCP assembly, or the overall regulation of virulence genes. Thus, His-tagged ToxT appears to retain its regulatory function with minimal impact under the tested conditions (Kim et al., 2017).

### Expression of native ToxT and His-tagged ToxT in the *V. cholerae* O395 strain

**Figure 1:**
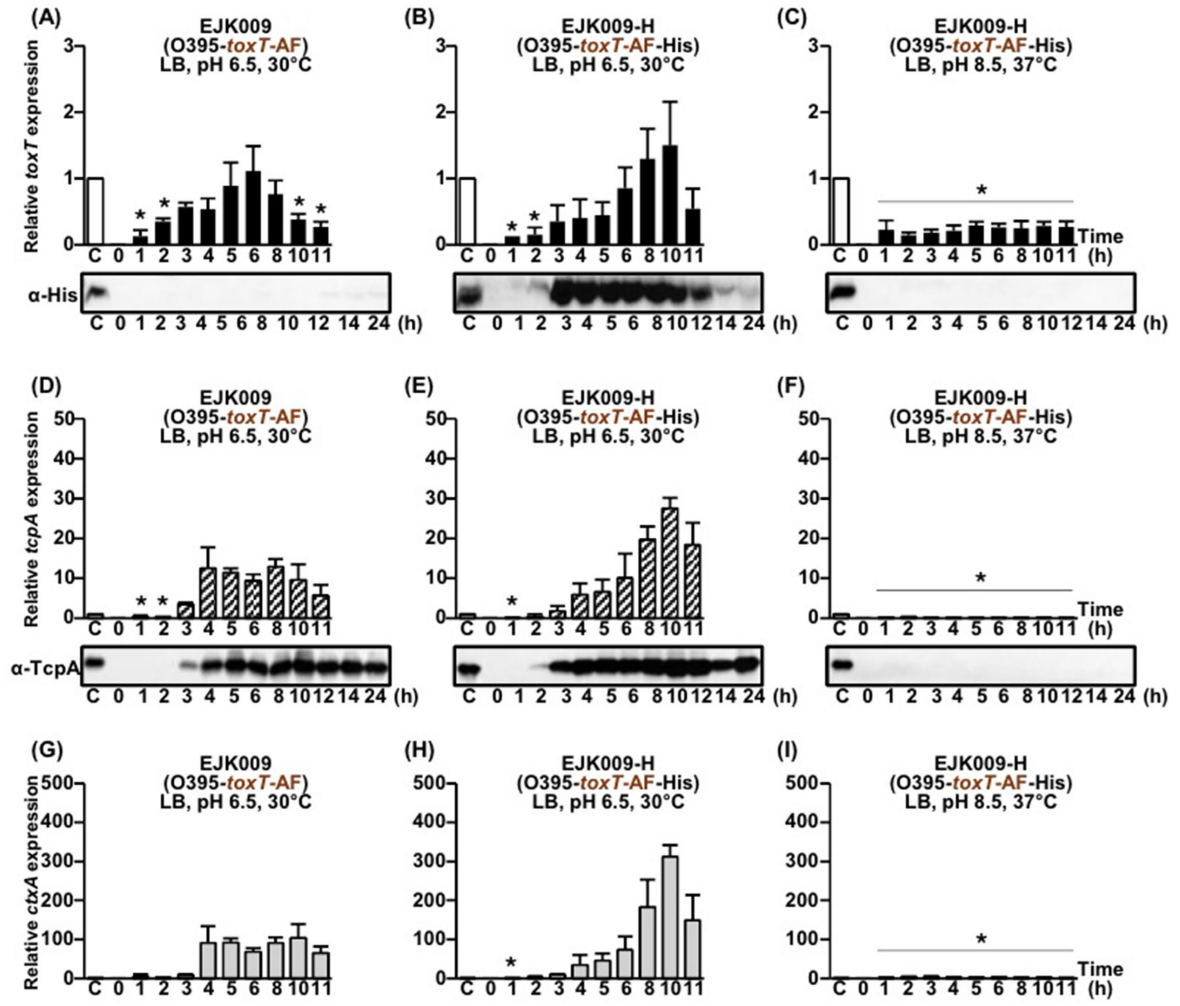
Comparison of native and His-tagged *toxT*, *tcpA*, and *ctxAB* expression in isogenic derivatives EJK009 (O395-*toxT*-AF) and EJK009-H (O395-*toxT*-AF-His). **(A–C)** *toxT* expression was analyzed by qRT-PCR, and His-tagged ToxT protein was detected by Western blot using anti-His-tag antibodies. **(A)** Native *toxT* mRNA in EJK009 cultured in LB medium (pH 6.5) at 30°C; **(B)** His-tagged *toxT* mRNA in EJK009-H cultured in LB medium (pH 6.5) at 30°C; and **(C)** His-tagged *toxT* mRNA in EJK009-H cultured in LB medium (pH 8.5) at 37°C. **(D–F)** *tcpA* expression was assessed by qRT-PCR, and TcpA protein levels were examined by Western blot using anti-TcpA antibodies: **(D)** EJK009 cultured in LB (pH 6.5) at 30°C; **(E)** EJK009-H under the same conditions; and **(F)** EJK009-H cultured in LB (pH 8.5) at 37°C. **(G–I)** qRT-PCR analysis of *ctxAB* mRNA: **(G)** EJK009 and **(H)** EJK009-H cultured in LB medium (pH 6.5) at 30°C; **(I)** EJK009-H cultured in LB medium (pH 8.5) at 37°C. All qRT-PCR expression values were normalized to the housekeeping gene *gyrA*. The relative expression levels of *toxT* (black bars), *tcpA* (diagonal bars), and *ctxAB* (gray bars) at each time point are shown. The *toxT*, *tcpA*, and *ctxAB* expression levels in the 4 h culture of O395-H (O395-*toxT*-SY-His, shown as lane C, white bars) were set to 1 for normalization. Statistical significance was assessed by one-way ANOVA followed by Dunnett’s multiple-comparison test using the 4 h O395-H condition as the reference. Only conditions that showed statistically significant decreases relative to the reference are indicated with asterisks (*p* < 0.05); conditions that were not significantly different from or higher than the reference are not annotated.

All four native *toxT* alleles (*toxT*-SY, *toxT*-SF, *toxT*-AY, and *toxT*-AF) have been shown to activate virulence gene expression under virulence-inducing conditions in the classical biotype strain O395 (Lee et al., 2023; Choi et al., 2023). To assess the functionality of native and His-tagged *toxT* variants, we compared the expression of *toxT* at the mRNA level by RT-PCR and at the protein level using Western blotting (anti-His-tag) in isogenic derivatives expressing the *toxT*-AF allele, with (EJK009-H) or without (EJK009) a His-tag, across different growth phases (Figure 1). These isogenic derivatives were cultured under two conditions: agglutinating conditions (30°C in LB medium, pH adjusted to 6.5) and virulence-repressing conditions (LB medium at 37°C, pH adjusted to 8.5).

As a baseline, expression levels in O395-H (His-tagged *toxT-SY*) were used for normalization, since in the parental O395 strain TcpA first became detectable by Western blot after 4 h under agglutinating conditions. At this reference point, transcript levels of *toxT, tcpA,* and *ctxAB* in O395-H were set to 1 (100%), and the relative expression of these genes was compared across strains carrying different *toxT* alleles.

Under virulence-inducing culture conditions, EJK009 (*toxT*-AF) and EJK009-H (His-tagged *toxT*-AF) displayed very similar timing and magnitude of virulence gene expression, with only minor variation (Figures 1A,B). *toxT* transcripts were detectable as early as 2–3 h post-inoculation in both strains, with expression peaking at approximately 6–8 h. Consistent with transcript levels, ToxT protein was detectable by Western blot analysis as early as 3 h post-cultivation. Under virulence-inducing conditions, ToxT protein levels peaked between 3 and 6 h, gradually decreasing thereafter, with nearly undetectable levels after 14 h (Figure 1B). Under virulence-repressing conditions, only minimal levels of *toxT* mRNA were detected, and no detectable ToxT protein was observed (Figure 1C).

Taken together, these results indicate that His-tagged *toxT* constructs closely approximate the expression dynamics of native *toxT*, supporting their use as reliable surrogates for studying virulence gene regulation in *V. cholerae* (Lee et al., 2023). As described below, the expression patterns of *tcpA* were comparable between His-tagged and native *toxT* alleles across different strain backgrounds (summarized in Supplementary Table S4).

### Virulence gene expression by native *toxT*-AF and His-tagged *toxT*-AF in the O395 strain

Based on the observation that His-tagged *toxT*-AF exhibited expression levels comparable to those of native *toxT*-AF, we next investigated whether both native ToxT-AF and His-tagged ToxT-AF similarly activated the expression of downstream target genes, *tcpA* and *ctxAB*. The expression of these target genes appeared to follow a slight delay relative to *toxT* expression.

Under virulence-inducing culture conditions, *tcpA* and *ctxAB* mRNA became detectable approximately 3–4 h after cultivation (Figures 1D,E,G,H), with TcpA protein also first detected at this time. In the His-tagged variant, a modest delay was observed in the peak expression of *tcpA* and *ctxAB*. TcpA protein levels were maintained up to 24 h in both isogenic derivatives (Figures 1D,E). In contrast, under virulence-repressing conditions, no expression of *tcpA* or *ctxAB* was detected (Figures 1F,I).

These results suggest that the addition of a His-tag does not alter the capacity of ToxT-AF to activate transcription of virulence genes under the tested conditions.

### Virulence gene expression by alternative *toxT* alleles in the O395 strain

Given that the expression levels of His-tagged *toxT*-AF—and consequently *tcpA* and *ctxAB*—were comparable to those of native *toxT*-AF in the O395 strain, we reasoned that the virulence gene expression pattern in isogenic derivatives carrying the His-tagged *toxT* allele could serve as a proxy for those carrying the corresponding native *toxT* allele. On this basis, we proceeded to investigate virulence gene expression in isogenic derivatives harboring His-tagged *toxT* alleles.

**Figure 2:**
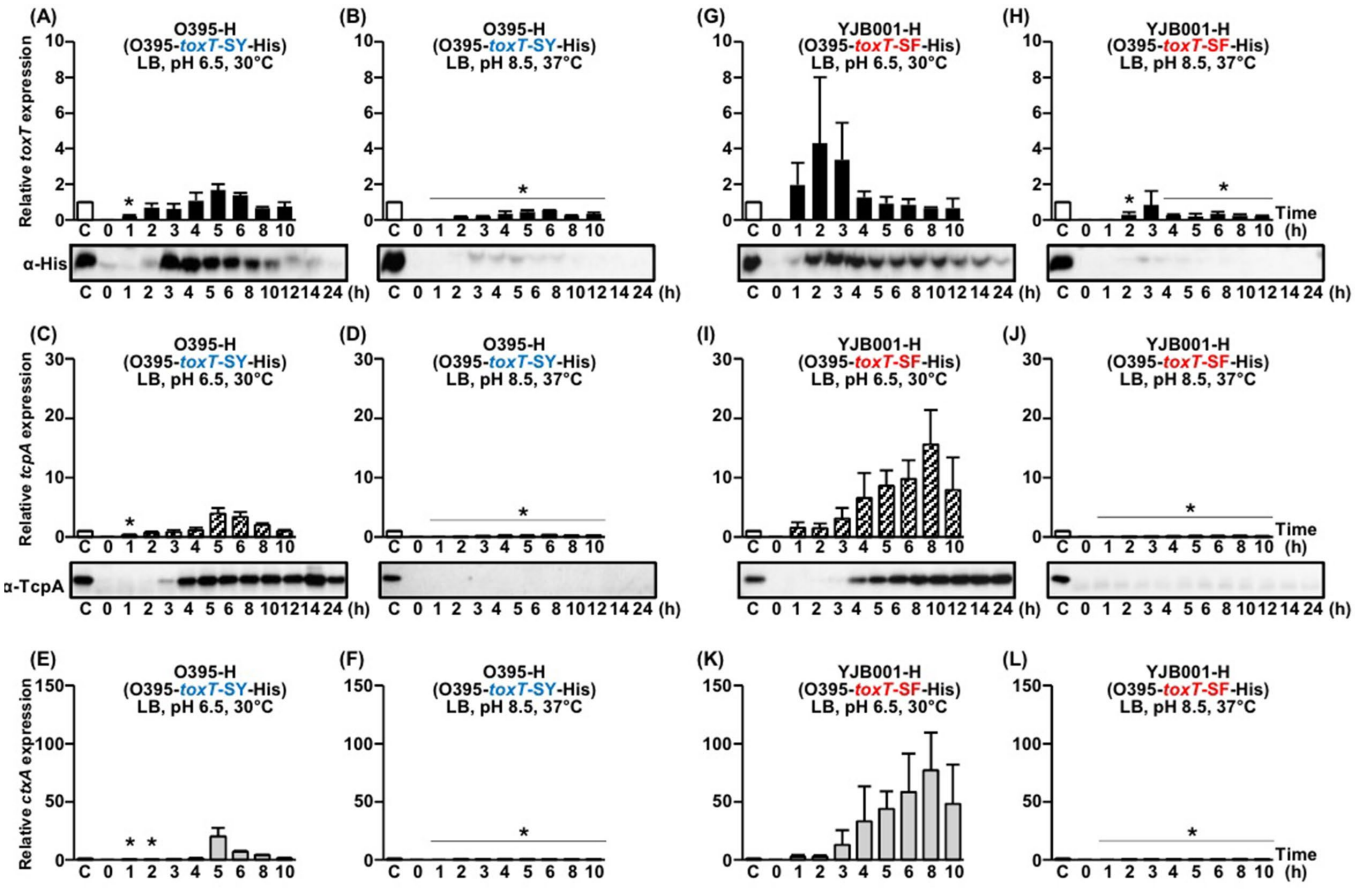
Expression of His-tagged *toxT*, *tcpA*, and *ctxAB* in isogenic derivatives O395-H (O395-*toxT*-SY-His) and YJB001-H (O395-*toxT*-SF-His). **(A–F)** O395-H: **(A,B)** qRT-PCR analysis of His-tagged *toxT* mRNA and Western blot detection of ToxT using anti-His-tag antibodies in cultures grown in LB medium at **(A)** pH 6.5, 30°C and **(B)** pH 8.5, 37°C. **(C,D)** qRT-PCR analysis of *tcpA* mRNA and Western blot detection of TcpA using anti-TcpA antibodies under the same respective conditions. **(E,F)** qRT-PCR analysis of *ctxAB* mRNA in O395-H cultured in LB medium at **(E)** pH 6.5, 30°C and **(F)** pH 8.5, 37°C. **(G–L)** YJB001-H: **(G,H)** qRT-PCR analysis of His-tagged *toxT* mRNA and Western blot detection of ToxT using anti-His-tag antibodies in cultures grown in LB medium at **(G)** pH 6.5, 30°C and **(H)** pH 8.5, 37°C. **(I,J)** qRT-PCR analysis of *tcpA* mRNA and Western blot detection of TcpA under the same respective conditions. **(K,L)** qRT-PCR analysis of *ctxAB* mRNA in YJB001-H cultured in LB medium at **(K)** pH 6.5, 30°C and **(L)** pH 8.5, 37°C. All qRT-PCR expression values were normalized to the housekeeping gene *gyrA*. Relative expression levels of His-tagged *toxT* (black bars), *tcpA* (diagonal bars), and *ctxAB* (gray bars) are shown for each time point. For normalization, the expression level of each gene in the 4 h culture of O395-H grown in LB medium (pH 6.5) at 30°C was set to 1 (lane C, white bar). Statistical significance was assessed by one-way ANOVA followed by Dunnett’s multiple-comparison test using the 4 h O395-H condition as the reference. Only conditions that showed statistically significant decreases relative to the reference are indicated with asterisks (*p* < 0.05); conditions that were not significantly different from or higher than the reference are not annotated.

*toxT*-SY: The O395 strain harbors the *toxT*-SY allele. Under virulence-inducing culture conditions, *toxT* mRNA in O395-H (His-tagged *toxT*-SY) was detectable within 2 h of cultivation, reaching its highest levels at 5 h (Figure 2A). His-tagged ToxT protein was first observed at 3 h, with protein levels peaking between 3 and 4 h (Figure 2A). Both *toxT* mRNA and ToxT protein levels began to decline after reaching their peaks, with *toxT* mRNA decreasing by 8 h and ToxT protein levels dropping by 12 h. Under virulence-repressing conditions, *toxT* mRNA levels were minimal, and ToxT protein production was negligible (Figure 2B).

The expression of *tcpA* and *ctxAB* mRNA was first detected between 3 and 4 h after cultivation, with peak expression occurring at approximately 5–6 h, followed by a gradual decrease (Figures 2C,E). TcpA protein levels became detectable starting at 4 h, and remained detectable up to 24 h post-cultivation (Figure 2C). No expression of *tcpA* and *ctxAB* was detected under virulence-repressing conditions (Figures 2D,F).

*toxT*-SF: The expression of His-tagged *toxT*-SF in YJB001-H under virulence-inducing conditions was first detectable within 1 h of culture, peaking at 2 h before gradually decreasing (Figure 2G). His-tagged ToxT was detectable starting at 2 h, with peak expression occurring between 2 and 4 h (Figure 2G). In contrast, under virulence-repressing conditions, *toxT* mRNA levels were minimal, and His-tagged ToxT was negligible (Figure 2H).

The mRNAs of *tcpA* and *ctxAB* were first detected at 3 h post-cultivation, reaching peak levels at 8 h, followed by a decline (Figures 2I,K). TcpA protein levels were first detectable at 4 h and TcpA protein levels remained substantial for up to 24 h (Figure 2I). Under virulence-repressing conditions, no expression of *tcpA* or *ctxAB* was observed (Figures 2J,L).

*toxT*-AY: The expression of His-tagged *toxT*-AY mRNA in EJK008-H (His-tagged *toxT*-AY) was first detected at 2 h post-cultivation under agglutinating conditions, with mRNA levels reaching a peak at 5 h before progressively declining (Supplementary Figure S4A). His-tagged ToxT protein was similarly detected starting at 2 h (Supplementary Figure S4A). Under virulence-repressing conditions, both *toxT*-AY mRNA and His-tagged ToxT protein were present at negligible levels (Supplementary Figure S4B).

Expression of the downstream targets *tcpA* and *ctxAB* was observed beginning at 2 h post-cultivation, peaking at 4 h before gradually declining (Supplementary Figures S4C,E). TcpA protein was first detected at 3 h and remained at substantial levels up to 24 h (Supplementary Figure S4C). Under virulence-repressing conditions, the expression of *tcpA* and *ctxAB* was negligible (Supplementary Figures S4D,F).

### Virulence gene expression in El Tor biotype strains

We investigated two *V. cholerae* El Tor biotype strains: the Wave 1 prototype strain N16961 and the Wave 3 atypical El Tor strain IB5230 (Mutreja et al., 2011; Chin et al., 2011). El Tor biotype strains typically express virulence genes under AKI conditions but not when cultured in LB medium (Lee et al., 2023; Kim et al., 2022; Lee et al., 2024). In previous work, we showed that introducing alternative *toxT* alleles (*toxT*-AY and *toxT*-AF) into N16961 enabled the strain to express virulence genes in aerated LB medium, even without pH adjustment to 6.5 (Lee et al., 2023; Baek et al., 2020). In the present study, we monitored virulence gene expression in El Tor biotype strains cultured in LB medium without pH adjustment, to assess whether allele-specific or strain-specific regulation could drive virulence gene induction under these otherwise non-inducing conditions.

### N16961

The *V. cholerae* N16961 strain carries the authentic *toxT*-SY allele but does not exhibit virulence gene expression under LB culture conditions (Lee et al., 2023; Lee et al., 2024). An isogenic derivative in which *toxT*-SY was replaced with *toxT*-SF similarly failed to induce virulence gene expression (Lee et al., 2023). In contrast, previous studies have shown that substitution with either *toxT*-AY or *toxT*-AF enabled virulence gene expression in LB medium at 30°C (Lee et al., 2023; Choi et al., 2023; Lee et al., 2024).

**Figure 3:**
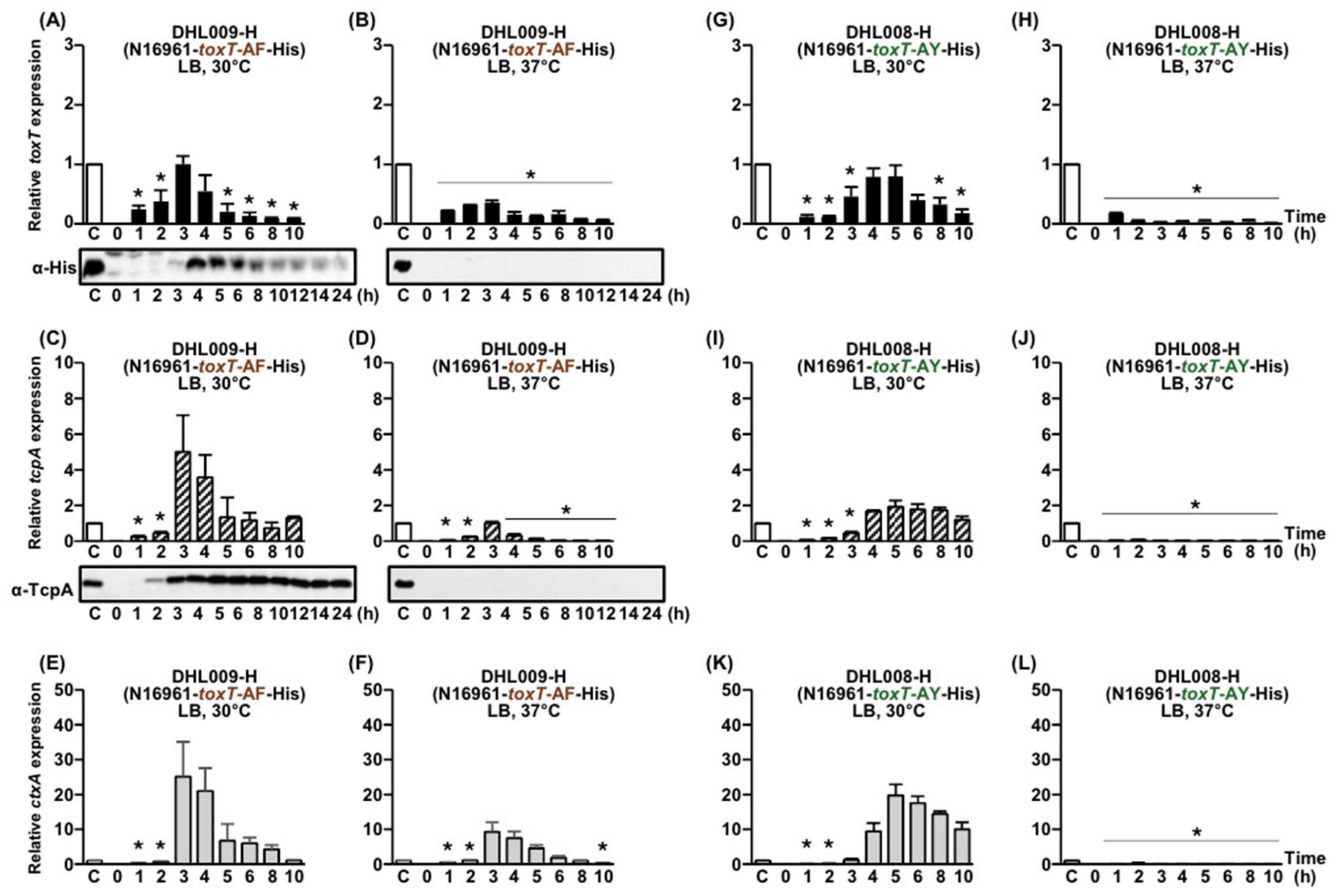
Comparison of His-tagged *toxT*, *tcpA*, and *ctxAB* expression in DHL009-H (N16961-*toxT*-AF-His) and DHL008-H (N16961-*toxT*-AY-His). **(A–F)** DHL009-H: **(A,B)** qRT-PCR analysis of His-tagged *toxT* mRNA and Western blot detection of ToxT using anti-His-tag antibodies in cultures grown in LB medium at **(A)** 30°C and **(B)** 37°C. **(C,D)** qRT-PCR analysis of *tcpA* mRNA and Western blot detection of TcpA using anti-TcpA antibodies under the same respective conditions. **(E,F)** qRT-PCR analysis of *ctxAB* mRNA in DHL009-H cultured in LB medium at **(E)** 30°C and **(F)** 37°C. **(G–L)** DHL008-H: qRT-PCR analysis of His-tagged *toxT* mRNA in cultures grown in LB medium at **(G)** 30°C and **(H)** 37°C. **(I,J)** qRT-PCR analysis of *tcpA* mRNA under the same respective conditions. **(K,L)** qRT-PCR analysis of *ctxAB* mRNA in DHL008-H cultured in LB medium at **(K)** 30°C and **(L)** 37°C. All qRT-PCR expression values were normalized to the housekeeping gene *gyrA*. Relative expression levels of His-tagged *toxT* (black bars), *tcpA* (diagonal bars), and *ctxAB* (gray bars) are shown for each time point. The expression level of each gene in the 4-h culture of O395-H grown in LB medium (pH 6.5) at 30°C was set to 1 (lane C, white bar) and used for normalization. Statistical significance was assessed by one-way ANOVA followed by Dunnett’s multiple-comparison test using the 4 h O395-H condition as the reference. Only conditions that showed statistically significant decreases relative to the reference are indicated with asterisks (*p* < 0.05); conditions that were not significantly different from or higher than the reference are not annotated.

In this study, we investigated virulence gene expression in isogenic derivatives of N16961 carrying four different His-tagged *toxT* alleles. The isogenic derivatives carrying His-tagged *toxT*-AF (DHL009-H) or His-tagged *toxT*-AY (DHL008-H) exhibited virulence gene expression during the early exponential phase at 30°C (Figures 3A,G). Once mRNA transcription was initiated, ToxT protein was detectable shortly after transcript initiation and remained detectable for approximately 12 h (Figures 3A,G). The expression patterns of the downstream targets *tcpA* and *ctxAB* followed a similar temporal profile (Figures 3C,E,I,K). Additionally, no expression of *tcpA* or *ctxAB* was detected at 37°C (Figures 3B,D,F,H,J,L).

**Figure 4:**
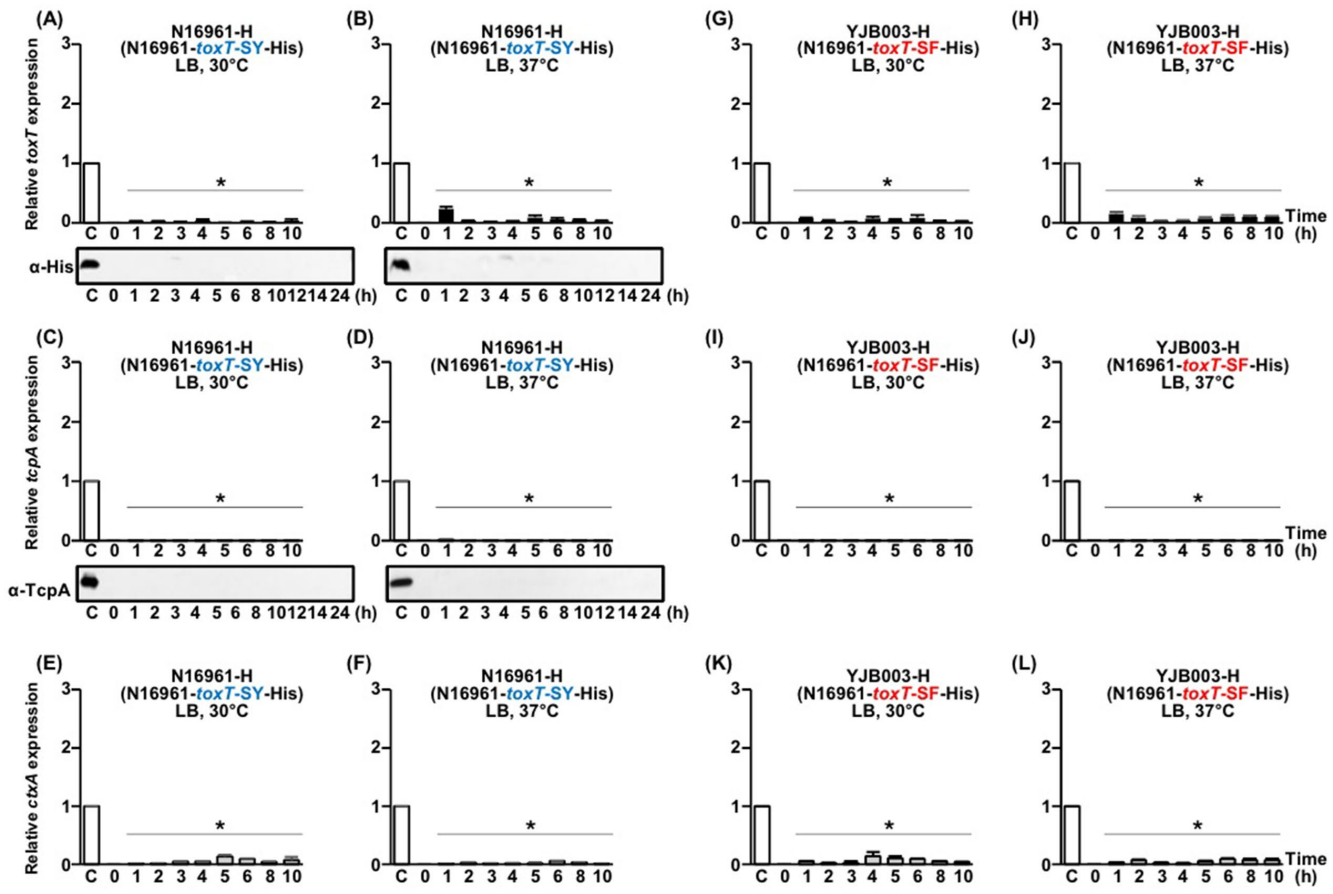
Comparison of His-tagged *toxT*, *tcpA*, and *ctxAB* expression in N16961-H (N16961-*toxT*-SY-His) and YJB003-H (N16961-*toxT*-SF-His). **(A–F)** N16961-H: **(A,B)** qRT-PCR analysis of His-tagged *toxT* mRNA and Western blot detection of ToxT using anti-His-tag antibodies in cultures grown in LB medium at **(A)** 30°C and **(B)** 37°C. **(C,D)** qRT-PCR analysis of *tcpA* mRNA and Western blot detection of TcpA using anti-TcpA antibodies under the same respective conditions. **(E,F)** qRT-PCR analysis of *ctxAB* mRNA in N16961-H cultured in LB medium at **(E)** 30°C and **(F)** 37°C. **(G–L)** YJB003-H: **(G,H)** qRT-PCR analysis of His-tagged *toxT* mRNA in cultures grown in LB medium at **(G)** 30°C and **(H)** 37°C. **(I,J)** qRT-PCR analysis of *tcpA* mRNA under the same respective conditions. **(K,L)** qRT-PCR analysis of *ctxAB* mRNA in YJB003-H cultured in LB medium at **(K)** 30°C and **(L)** 37°C. All qRT-PCR expression values were normalized to the housekeeping gene *gyrA*. Relative expression levels of His-tagged *toxT* (black bars), *tcpA* (diagonal bars), and *ctxAB* (gray bars) are shown for each time point. The expression level of each gene in the 4 h culture of O395-H grown in LB medium (pH 6.5) at 30°C was set to 1 (lane C, white bar) and used for normalization. Statistical significance was assessed by one-way ANOVA followed by Dunnett’s multiple-comparison test using the 4-h O395-H condition as the reference. Only conditions that showed statistically significant decreases relative to the reference are indicated with asterisks (*p* < 0.05); conditions that were not significantly different from or higher than the reference are not annotated.

By contrast, no virulence gene expression was observed in isogenic derivatives carrying His-tagged *toxT*-SY (N16961-H) or His-tagged *toxT*-SF (YJB003-H) alleles (Figures 4A–L), consistent with results obtained for the corresponding native alleles. Overall, *tcpA* and *ctxAB* expression patterns observed with His-tagged *toxT* alleles were in agreement with prior reports for native *toxT* alleles (Supplementary Table S4) (Lee et al., 2023; Lee et al., 2024).

### IB5230

The atypical El Tor strain *V. cholerae* IB5230 also carries the authentic *toxT*-SY allele. Unlike N16961, IB5230 expressed virulence genes with either the *toxT*-SY or *toxT*-SF allele. In contrast, replacement of *toxT*-SY allele with *toxT*-AY or *toxT*-AF allele ab prevented detectable virulence gene expression (Lee et al., 2023; Lee et al., 2024).

**Figure 5:**
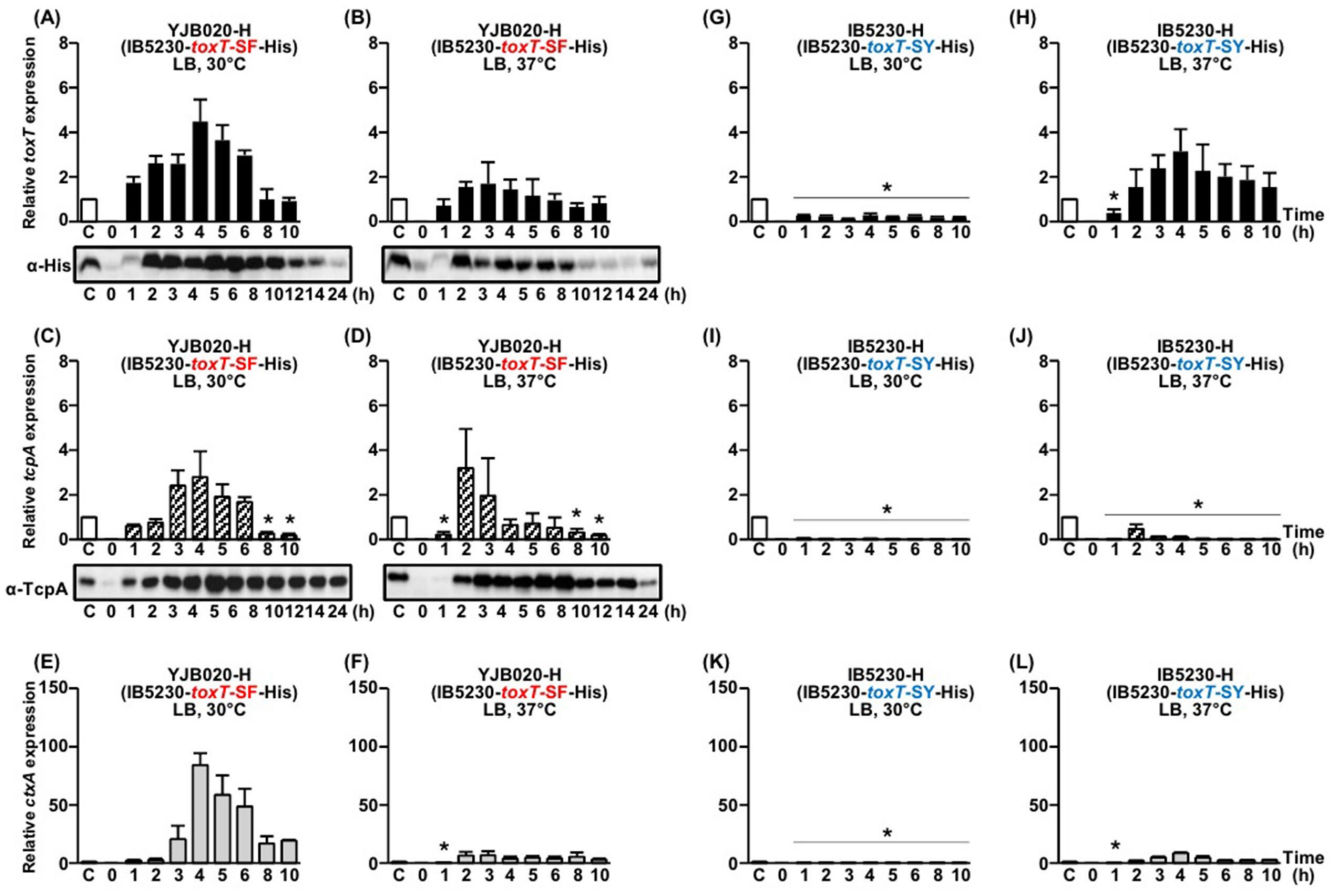
Comparison of His-tagged *toxT*, *tcpA*, and *ctxAB* expression in YJB020-H (IB5230-*toxT*-SF-His) and IB5230-H (IB5230-*toxT*-SY-His). **(A–F)** YJB020-H: **(A,B)** qRT-PCR analysis of His-tagged *toxT* mRNA and Western blot detection of ToxT using anti-His-tag antibodies in cultures grown in LB medium at **(A)** 30°C and **(B)** 37°C. **(C,D)** qRT-PCR analysis of *tcpA* mRNA and Western blot detection of TcpA using anti-TcpA antibodies under the same respective conditions. **(E,F)** qRT-PCR analysis of *ctxAB* mRNA in YJB020-H cultured in LB medium at **(E)** 30°C and **(F)** 37°C. **(G–L)** IB5230-H: **(G,H)** qRT-PCR analysis of His-tagged *toxT* mRNA in cultures grown in LB medium at **(G)** 30°C and **(H)** 37°C. **(I,J)** qRT-PCR analysis of *tcpA* mRNA under the same respective conditions. **(K,L)** qRT-PCR analysis of *ctxAB* mRNA in IB5230-H cultured in LB medium at **(K)** 30°C and **(L)** 37°C. All qRT-PCR expression values were normalized to the housekeeping gene *gyrA*. Relative expression levels of His-tagged *toxT* (black bars), *tcpA* (diagonal bars), and *ctxAB* (gray bars) are shown for each time point. The expression level of each gene in the 4-h culture of O395-H grown in LB medium (pH 6.5) at 30°C was set to 1 (lane C, white bar) and used for normalization. Statistical significance was assessed by one-way ANOVA followed by Dunnett’s multiple-comparison test using the 4 h O395-H condition as the reference. Only conditions that showed statistically significant decreases relative to the reference are indicated with asterisks (*p* < 0.05); conditions that were not significantly different from or higher than the reference are not annotated.

YJB020-H (His-tagged *toxT*-SF) expressed virulence genes at both 30°C and 37°C although *ctxAB* expression was restricted to 30°C. At 30°C, *toxT* mRNA became detectable starting 1 h after inoculation, reaching peak levels at approximately 4–5 h (Figure 5A). ToxT protein became detectable between 1 and 2 h, with substantial levels maintained for approximately 10 h. Expression of *tcpA* and *ctxAB* followed shortly after *toxT* expression (Figures 5C,E).

At 37°C, *toxT* mRNA was detectable as early as 1 h after inoculation, reaching its peak at approximately 3–4 h. ToxT protein also appeared between 1 and 2 h but declined more rapidly than under the 30°C condition (Figure 5B). The expression of *tcpA* and *ctxAB* appeared to be differentially regulated at 37°C. While *tcpA* mRNA and TcpA protein were detected (Figure 5D), *ctxAB* expression was not observed (Figure 5F). These findings were consistent with results from YJB020 carrying the native *toxT*-SF allele (Lee et al., 2023; Lee et al., 2024).

In the isogenic derivative of IB5230 harboring the His-tagged *toxT*-SY allele (IB5230-H), a temperature-dependent shift in *toxT* and virulence gene expression was observed. At 30°C, no expression of *toxT, tcpA*, or *ctxAB* was detected (Figures 5G,I,K). In contrast, when cultured at 37°C, both *toxT* and *ctxAB* were expressed; however, *tcpA* expression remained absent under these conditions (Figures 5H,J,L).

**Figure 6:**
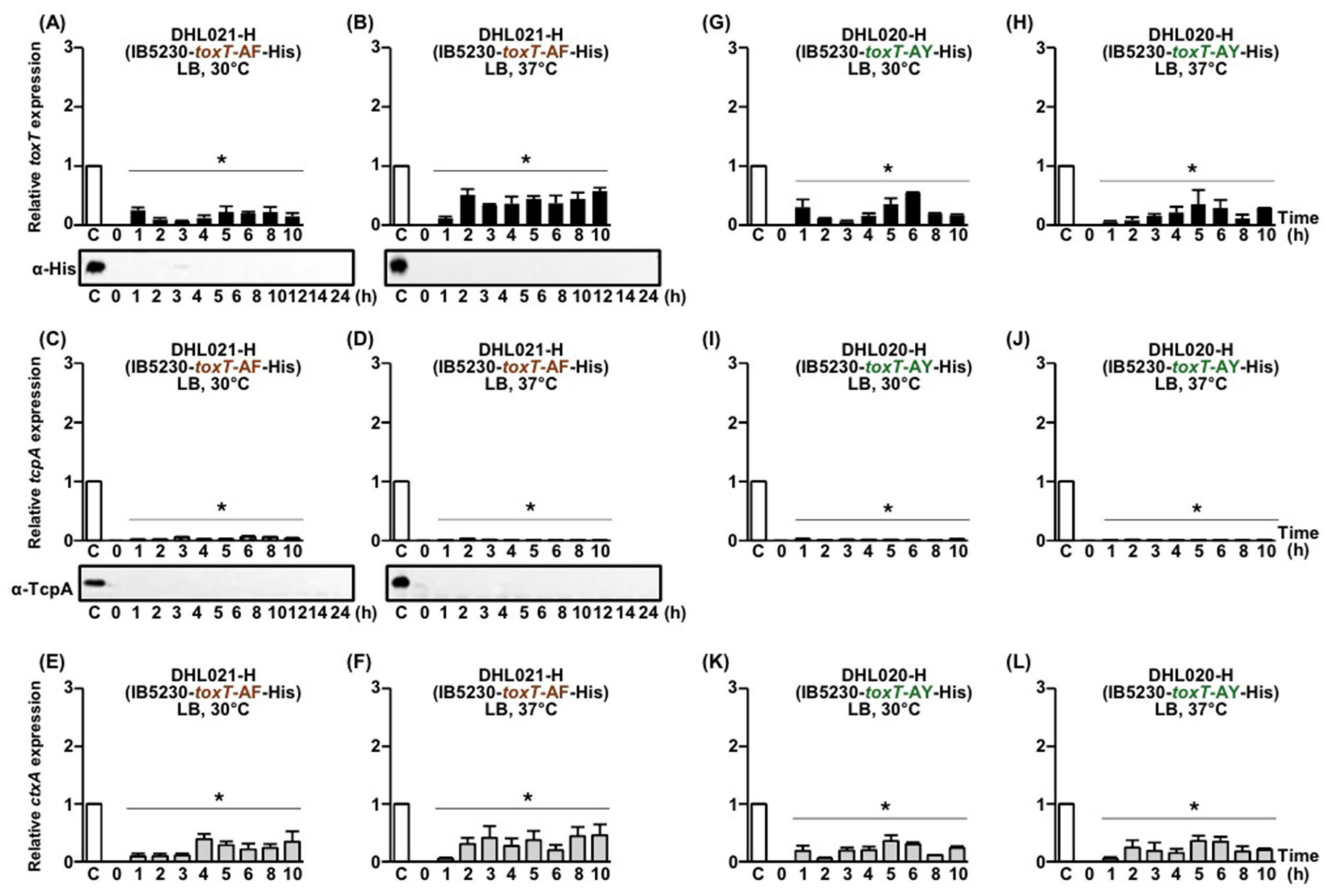
Expression of His-tagged *toxT*, *tcpA*, and *ctxAB* in DHL021-H (IB5230-*toxT*-AF-His) and DHL020-H (IB5230-*toxT*-AY-His). **(A–F)** DHL021-H: **(A,B)** qRT-PCR analysis of His-tagged *toxT* mRNA and Western blot detection of ToxT using anti-His-tag antibodies in cultures grown in LB medium at **(A)** 30°C and **(B)** 37°C. **(C,D)** qRT-PCR analysis of *tcpA* mRNA and Western blot detection of TcpA using anti-TcpA antibodies under the same respective conditions. **(E,F)** qRT-PCR analysis of *ctxAB* mRNA in DHL021-H cultured in LB medium at **(E)** 30°C and **(F)** 37°C. **(G–L)** DHL020-H: **(G,H)** qRT-PCR analysis of His-tagged *toxT* mRNA in cultures grown in LB medium at **(G)** 30°C and **(H)** 37°C. **(I,J)** qRT-PCR analysis of *tcpA* mRNA under the same respective conditions. **(K,L)** qRT-PCR analysis of *ctxAB* mRNA in DHL020-H cultured in LB medium at **(K)** 30°C and **(L)** 37°C. All qRT-PCR expression values were normalized to the housekeeping gene *gyrA*. Relative expression levels of His-tagged *toxT* (black bars), *tcpA* (diagonal bars), and *ctxAB* (gray bars) are shown for each time point. The expression level of each gene in the 4 h culture of O395-H grown in LB medium (pH 6.5) at 30°C was set to 1 (lane C, white bar) and used for normalization. Statistical significance was assessed by one-way ANOVA followed by Dunnett’s multiple-comparison test using the 4 h O395-H condition as the reference. Only conditions that showed statistically significant decreases relative to the reference are indicated with asterisks (*p* < 0.05); conditions that were not significantly different from or higher than the reference are not annotated.

The isogenic derivative of IB5230 harboring the His-tagged *toxT*-AF allele (DHL021-H) did not exhibit *toxT*, *tcpA*, or *ctxAB* expression at either 30°C or 37°C (Figures 6A–F). Similarly, in DHL020-H, which harbors the His-tagged *toxT*-AY allele, expression of *toxT*, *tcpA*, and *ctxAB* remained negligible under both temperature conditions (Figures 6G–L).

In IB5230 derivatives carrying His-tagged toxT alleles, the *tcpA* and *ctxAB* expression patterns observed—whether expression was detected at 37°C or showed differential regulation—closely paralleled those obtained with the corresponding native *toxT* alleles (Lee et al., 2023). These results indicate that His-tagging did not alter allele-specific regulatory outcomes and further highlight a strain-specific mode of virulence gene regulation in IB5230.

## Discussion

The primary virulence genes of *V. cholerae*, *ctxAB* and *tcpA*, are regulated by the ToxR regulon, with ToxT functioning as the final transcriptional activator in this regulatory cascade (Dorman and Dorman, 2018). Mechanistic studies of virulence gene expression have been extensively conducted in both classical and El Tor biotype strains of *V. cholerae* (Dorman and Dorman, 2018; Son et al., 2011; DiRita et al., 1996; Withey and DiRita, 2006; Davies et al., 2012). To facilitate these investigations and to better understand the pathogenic mechanisms of *V. cholerae*, specific culture conditions such as AKI or pH-adjusted LB have been developed (Jonson et al., 1990; Cobaxin et al., 2014). However, these conditions are not universally applicable to all *V. cholerae* strains, as significant strain-dependent variability in virulence gene expression has been observed (Lee et al., 2023; Baek et al., 2020; Kim et al., 2022).

Previously, we demonstrated that replacing the authentic *toxT* allele with alternative *toxT* alleles can alter virulence gene expression in *V. cholerae* strains (Kim et al., 2015; Lee et al., 2023; Baek et al., 2020; Kim et al., 2022; Lee et al., 2020). Depending on the strain, certain *toxT* alleles promoted virulence gene expression, whereas others did not (Lee et al., 2023). In this study, we examined the strain-dependent expression of four *toxT* alleles in *V. cholerae* and their impact on virulence gene regulation (Raskin et al., 2020; Howard et al., 2019). To enable direct comparisons, we generated isogenic derivatives in which the native *toxT* allele was replaced with the corresponding His-tagged version.

In the classical biotype strain O395, all four His-tagged *toxT* alleles were expressed and activated virulence gene expression, although the degree of activation varied. In contrast, the El Tor biotype strains exhibited allele-specific regulation. In N16961, His-tagged *toxT*-AY and *toxT*-AF were expressed and capable of activating virulence gene transcription under aerated LB culture conditions at 30°C. By contrast, His-tagged *toxT*-SY and *toxT*-SF were not expressed, leading to the absence of virulence gene expression. The opposite pattern was observed in IB5230: His-tagged *toxT*-SY and *toxT*-SF were expressed and activated virulence gene transcription, while His-tagged *toxT*-AY and *toxT*-AF were not expressed, resulting in a failure to induce virulence gene expression.

Consistent with previous reports on virulence gene expression driven by alternative *toxT* alleles in *V. cholerae* (Lee et al., 2023; Lee et al., 2024), introduction of a His-tag to the chromosomal *toxT* did not alter allele-specific expression or activity. *toxT* alleles capable of inducing virulence gene expression did so regardless of the presence of the His-tag, while *toxT* alleles that were unable to induce virulence gene expression remained inactive irrespective of the presence of the His-tag. Thus, detection of His-tagged ToxT reliably reflects the behavior of native ToxT under the tested laboratory conditions.

Across all strains, when *toxT* was expressed, both *toxT* mRNA and ToxT protein were detectable from the early exponential phase and persisted through early stationary phase. This stability suggests that ToxT is relatively stable during active growth, with little fluctuation unless culture conditions change or inhibitory factors are introduced. Consistently, *tcpA* and *ctxAB* were rapidly induced following ToxT activation, leading to the production of functional TCP and CT.

In El Tor biotype strains, *toxT* alleles that failed to induce virulence gene expression were not detectably transcribed, resulting in no detectable ToxT protein and, consequently, no expression of *ctxAB* or *tcpA*. These results suggest that the functional outcomes of specific *toxT* alleles can differ despite identical culture conditions, and that their activation or repression is likely determined by strain-specific regulatory manner.

Although the classical biotype strain O395 activated virulence gene expression with all *toxT* alleles, this trait was not shared by all classical biotype strains. Strains such as 569B, Cairo48, and Cairo50 displayed *toxT* allele-specific virulence gene expression, similar to that observed in El Tor biotype strains. For example, in the 569B strain, the authentic *toxT*-AY allele supported virulence gene expression, as did the alternative *toxT*-AF allele. However, replacement with *toxT*-SY or *toxT*-SF failed to support virulence gene activation (Lee et al., 2023; Kim et al., 2022). Similarly, the Cairo48 and Cairo50 strains, both used in oral cholera vaccine (OCV) development, naturally harbor the *toxT*-SY allele and fail to express virulence genes under agglutinating culture conditions. However, virulence gene expression could be restored when *toxT*-SY is replaced with alternative alleles—*toxT*-SF and *toxT*-AY in Cairo48, and *toxT*-AY in Cairo50 (Lee et al., 2023; Kim et al., 2022). These findings suggest that O395’s ability to activate virulence genes irrespective of *toxT* allele type is a distinctive feature of this strain rather than a general property of classical biotype strains.

All four *toxT* alleles were capable of activating virulence gene expression in the classical biotype strain O395, indicating that ToxT function itself does not differ substantially among alleles. This was further supported by our results that all four *toxT* alleles, when expressed exogenously under the P_BAD_ promoter in an isogenic derivative of the N16961 strain lacking the authentic *toxT* gene, successfully induced *tcpA* and *ctxAB* expression. Taken together, our results suggest that allele- and strain-dependent differences in virulence gene expression largely reflect variation in *toxT* expression levels, rather than differences in the intrinsic transcriptional activator capacity of ToxT.

Previous studies on ToxT have largely focused on its biochemical properties, DNA-binding activity, and modulation by environmental signals such as bile components and bicarbonate, generally assuming that *toxT* is expressed once appropriate virulence-inducing conditions are met (Childers and Klose, 2007; Lowden et al., 2010; Thomson and Withey, 2014; Raskin et al., 2020; Howard et al., 2019). In contrast, our results indicate that a major determinant of virulence gene activation in *V. cholerae* is whether *toxT* itself is transcriptionally expressed in a given strain–allele context.

While the upstream molecular mechanisms responsible for this strain- and allele-dependent *toxT* regulation remain to be elucidated, the strain-specific activation or repression of particular *toxT* alleles is likely to involve a combination of cis-regulatory elements and trans-acting factors within the ToxR regulon or associated regulatory networks. Elucidating the precise genetic and regulatory determinants that govern allele- and strain-dependent *toxT* transcription will be a key step toward understanding strain-specific virulence regulation in *V. cholerae*.

Nevertheless, several observations from the present study provide insight into how such differential regulation might arise. Previous studies have shown that ToxT can autoregulate its own expression (Withey and DiRita, 2006; Dittmer and Withey, 2012), suggesting that an initial, low level of ToxT may be produced in response to environmental stimuli and, upon reaching a critical threshold—potentially through autoregulatory feedback—lead to full activation of the virulence regulon. The extent of *toxT* expression may therefore be modulated by strain-specific factors or subtle differences in autoregulatory dynamics. In several cases in which virulence gene expression was not detected, we observed very low levels of *toxT* mRNA and faint ToxT protein signals (e.g., Figures 2B,H), which may represent sub-threshold or basal expression. Whether such low-level expression represents an inherent feature of the ToxR regulon or corresponds to an early phase of ToxT-mediated autoregulation remains to be clarified through further investigation.

Notably, IB5230, a strain associated with the 2010 Haitian cholera outbreak, exhibited robust expression of *toxT* and virulence genes even at 37°C. This may reflect physiological conditions encountered in the human intestine, suggesting that strain-specific differences in *toxT* regulation could influence colonization efficiency and pathogenicity. Before the 1990s, prototype El Tor biotype strains—such as N16961—were the predominant *V. cholerae* lineages circulating globally. Since the early 1990s, however, they have been gradually displaced by atypical El Tor strains, including IB5230, which now dominate recent outbreaks (Kim et al., 2015; Ramamurthy et al., 2019). These strains also differ in their regulation of virulence genes under laboratory conditions. While N16961 does not express virulence genes in LB medium, IB5230 exhibits detectable *toxT* and downstream virulence gene expression even at 37°C.

Although the basis of this shift remains unclear, differences in *toxT* regulation may have contributed to the selective replacement of prototype El Tor by atypical El Tor strains. This possibility could be examined using animal infection models or host-mimicking systems that more closely reflect the intestinal environment. Further investigation is needed to clarify whether such regulatory differences are linked to changes in fitness, host adaptation, or transmission dynamics (Kim et al., 2015; Satchell et al., 2016; Ghosh et al., 2019).

The findings reported here were obtained under laboratory conditions and may not fully capture the complexity of the *in vivo* environment; nevertheless, they provide important insights into the regulatory mechanisms governing virulence gene expression in *V. cholerae*. Our results suggest that *toxT* allele- and strain-specific regulation is more intricate and finely controlled than previously appreciated. Further studies will be needed to establish whether the strain-dependent *toxT* activation patterns observed *in vitro* correspond to virulence gene expression and disease progression *in vivo*. Elucidating the genetic and environmental factors that shape *toxT* activation will be essential for developing a more comprehensive understanding of virulence regulation during infection.

## Data Availability

The datasets presented in this study can be found in online repositories. The names of the repository/repositories and accession number(s) can be found at: the datas that support the findings of this study are openly available in Figshare at https://doi.org/10.6084/m9.figshare.30536213.
