# Supplementary figures and images for: Strain-dependent toxT expression, rather than ToxT activity, governs virulence gene regulation in Vibrio cholerae
Source: Front Microbiol. 2026 Feb 19;17:1755947. doi: 10.3389/fmicb.2026.1755947 (PMC12960492; doi:10.3389/fmicb.2026.1755947)

Fig. S1.

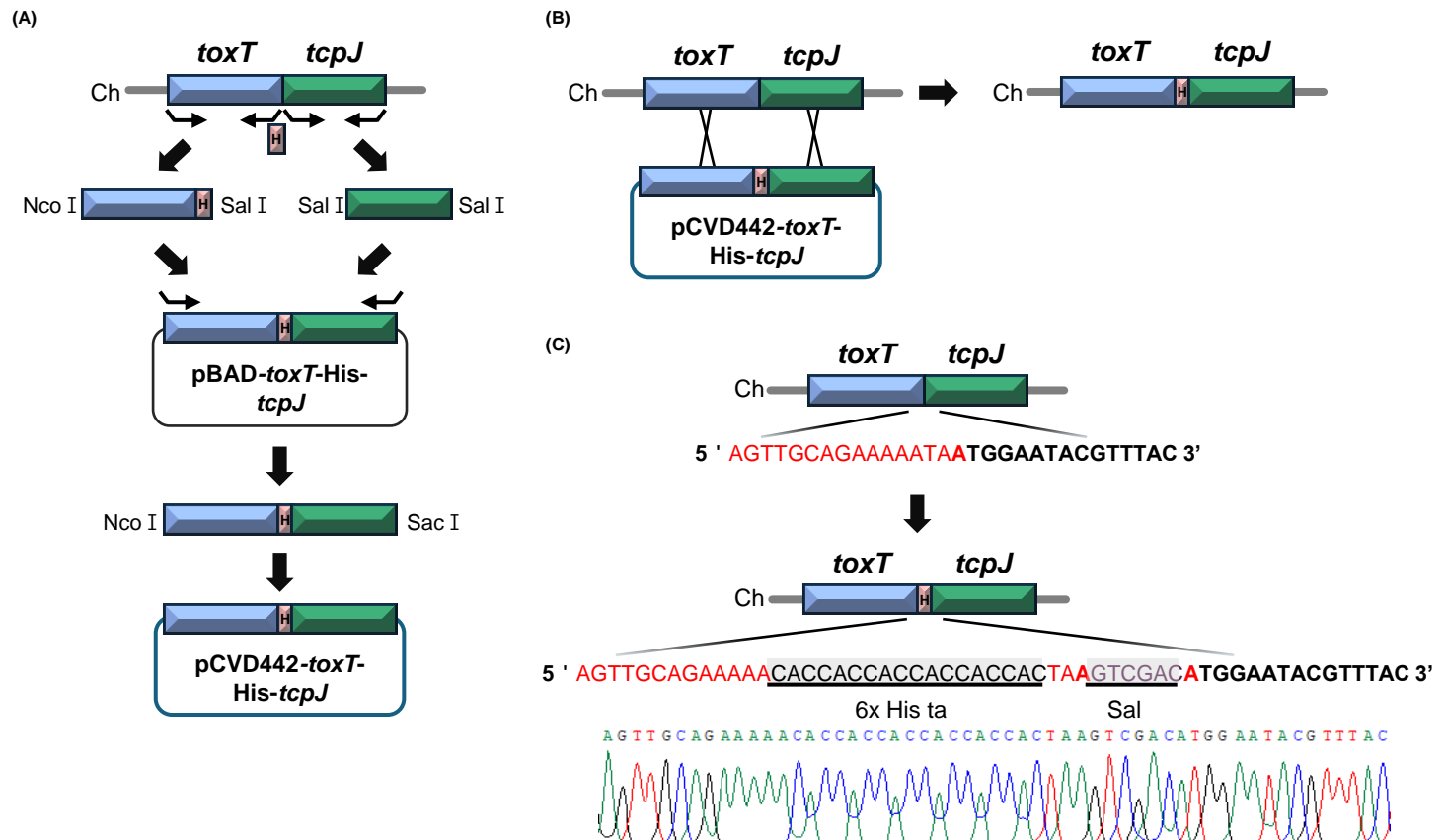

Supplement: Supplementary file 1 [file Data_Sheet_1.pdf]

Fig. S2

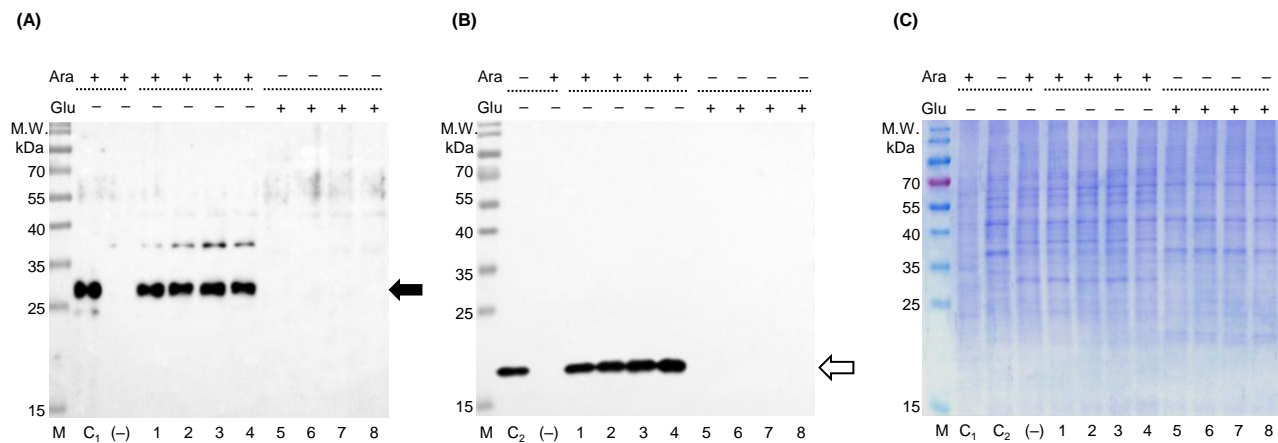

Supplement: Supplementary file 2 [file Data_Sheet_2.pdf]

Fig. S3

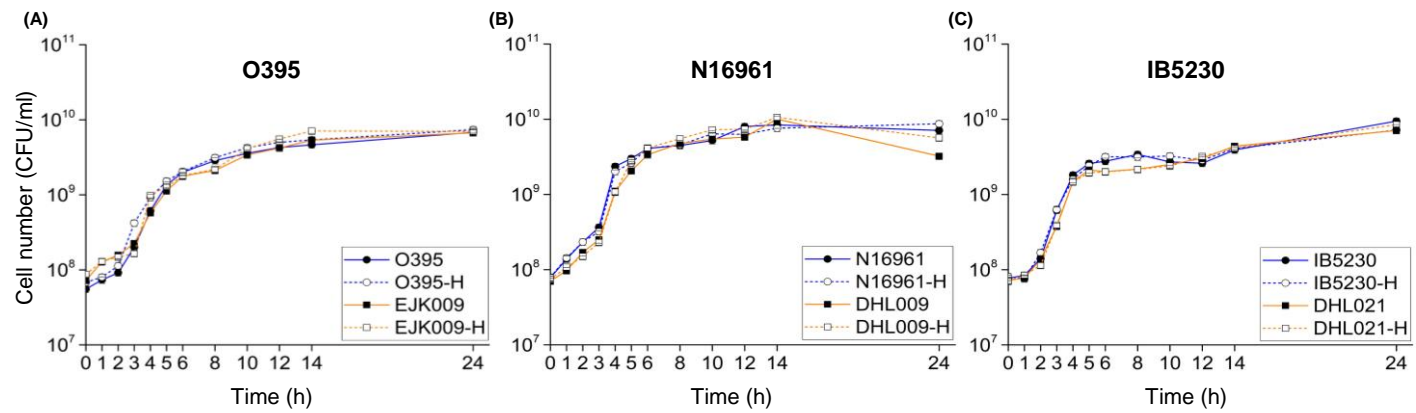

Supplement: Supplementary file 3 [file Data_Sheet_3.pdf]

Fig. S.4

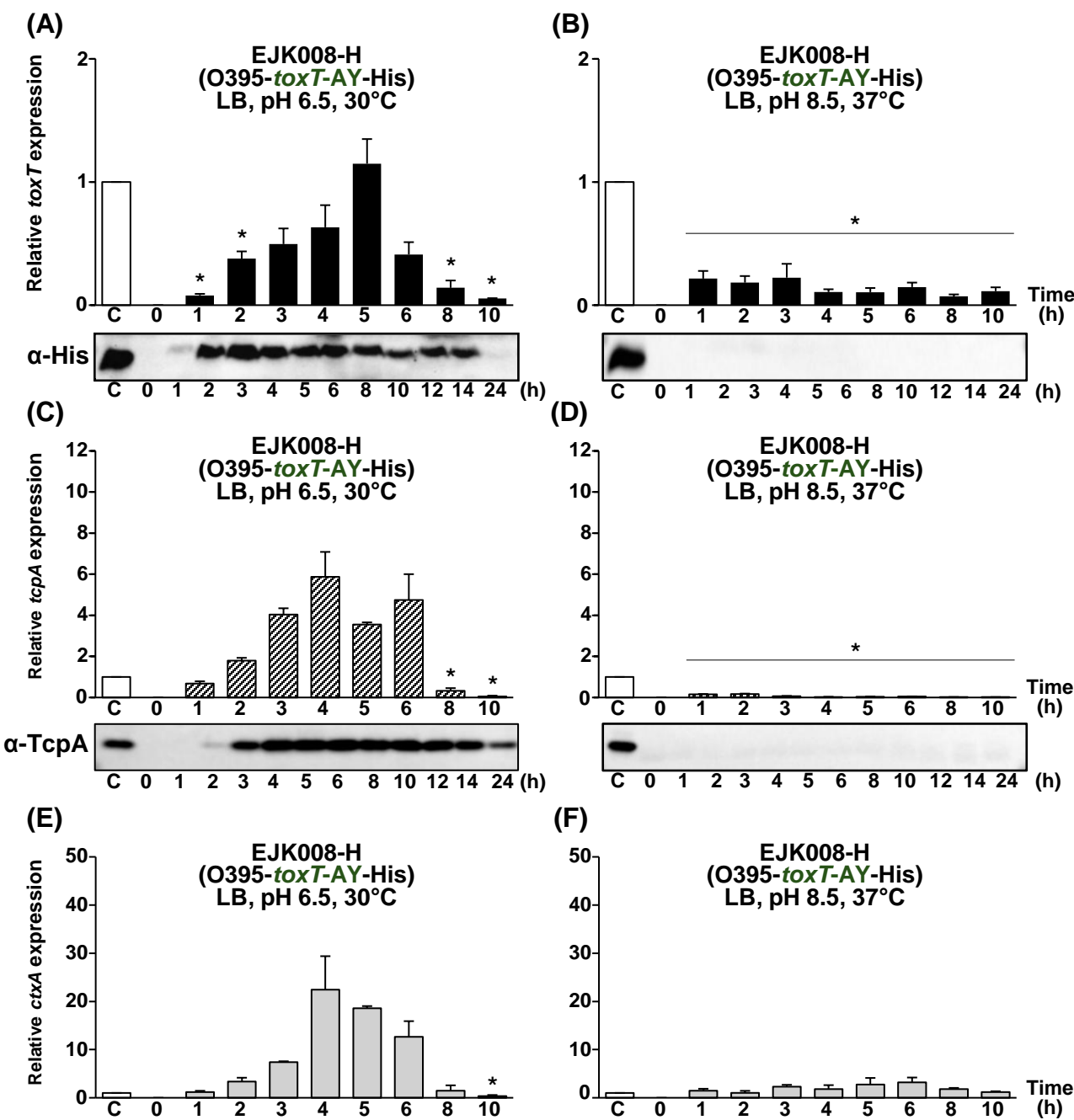

Supplement: Supplementary file 4 [file Data_Sheet_4.pdf]
