# Supplementary material for: Strain-dependent toxT expression, rather than ToxT activity, governs virulence gene regulation in Vibrio cholerae
Source: Front Microbiol. 2026 Feb 19;17:1755947. doi: 10.3389/fmicb.2026.1755947 (PMC12960492; doi:10.3389/fmicb.2026.1755947)
Supplement: Supplementary file 5 [file Table_1.docx]

**Table S1. *V. cholerae* strains and their isogenic derivatives.**

| **Strains** | ***toxT* genotype** | **Genome information and references** | **Strains** | ***toxT* genotype** | **Genome information and references** |
| --- | --- | --- | --- | --- | --- |
| Classical biotype |  |  | El Tor Biotype |  |  |
| O395 | O395-*toxT*-SY | CP000626/CP000627 (Mutreja et al., 2011) | N16961 | N16961-*toxT*-SY | AE003852/AE003853 (Heidelberg et al., 2000) |
| O395-H | O395-*toxT*-SY-His | This study | N16961-H | N16961-*toxT*-SY-His | This study |
| YJB001 | O395-*toxT*-SF | (Baek et al., 2020) | YJB003 | N16961-*toxT*-SF | (Baek et al., 2020) |
| YJB001-H | O395-*toxT*-SF-His | This study | YJB003-H | N16961-*toxT*-SF-His | This study |
| EJK008 | O395-*toxT*-AY | (Lee et al., 2023) | DHL008 | N16961-*toxT*-AY | (Lee et al., 2023) |
| EJK008-H | O395-*toxT*- AY-His | This study | DHL008-H | N16961-*toxT*-AY-His | This study |
| EJK009 | O395-*toxT*-AF | (Lee et al., 2023) | DHL009 | N16961-*toxT*-AF | (Lee et al., 2023) |
| EJK009-H | O395-*toxT*-AF-His | This study | DHL009-H | N16961-*toxT*-AF-His | This study |
| EJK010 | O395-Δ*toxT* | (Lee et al., 2023) | DHL010 | N16961-Δ*toxT* | (Lee et al., 2023) |
|  |  |  |  |  |  |
| 569B | 569B-*toxT*-AY | DADXPZ010000000 |  |  |  |
| EJK007 | 569B-*toxT*-AF | (Kim et al., 2022) | IB5230 | IB5230-*toxT*-SY | AELH00000000.1 (Chin et al., 2011) |
|  |  |  | IB5230-H | IB5230-*toxT*-SY-His | This study |
|  |  |  | YJB020 | IB5230-*toxT*-SF | (Baek et al., 2020) |
|  |  |  | YJB020-H | IB5230-*toxT*-SF-His | This study |
|  |  |  | DHL020 | IB5230-*toxT*-AY | (Lee et al., 2023) |
|  |  |  | DHL020-H | IB5230-*toxT*-AY-His | This study |
|  |  |  | DHL021 | IB5230-*toxT*-AF | (Lee et al., 2023) |
|  |  |  | DHL021-H | IB5230-*toxT*-AF-His | This study |
|  |  |  | DHL022 | IB5230-Δ*toxT* | (Lee et al., 2023) |

*toxT*-SY: *toxT* allele with Ser at position 65 and Tyr at position 139.

*toxT*-SF: *toxT* allele with Ser at position 65 and Phe at position 139.

*toxT*-AY: *toxT* allele with Ala at position 65 and Tyr at position 139.

*toxT*-AF: *toxT* allele with Ala at position 65 and Phe at position 139.
