# Supplementary material for: Strain-dependent toxT expression, rather than ToxT activity, governs virulence gene regulation in Vibrio cholerae
Source: Front Microbiol. 2026 Feb 19;17:1755947. doi: 10.3389/fmicb.2026.1755947 (PMC12960492; doi:10.3389/fmicb.2026.1755947)
Supplement: Supplementary file 6 [file Table_2.docx]

**Table S2. Oligonucleotide sequences for isogenic derivative construction and qRT-PCR.**

| **Primer** | **Sequence** | **Reference** |
| --- | --- | --- |
| **Primers for construction of isogenic derivatives of *V. cholerae* strains** | | |
| toxT-XbaIF | CCG GCC TCT AGA TAC GTG GAT GGC TCT CTG CG | (Kim et al., 2017) |
| toxT-SacIR | CCG GCC GAG CTC CAC TTG GTG CTA CAT TCA |  |
| TcpF-XbaIF | GGG TCT AGA GAA TTA AGT AAG CAC GGG TA | (Baek et al., 2020) |
| toxT-SacIR | CCG GCC GAG CTC CAC TTG GTG CTA CAT TCA |  |
| TcpF-KpnIF | GGG GGT ACC GAA TTA AGT AAG CAC GGG TA | (Lee et al., 2023) |
| TcpF-BamHIR | CCC GGA TCC TGC AAT TCC ACT ATC TAT CCA |  |
| TcpJ-BamHIF | GGG GGA TCC CTA GAG TCT CGA GGA GTA AAG | (Lee et al., 2023) |
| TcpJ-PstIR | CCC CTG CAG GCT GCA GAT AAA TAA ATG CC |  |
| TcpF-XbaIF | GGG TCT AGA GAA TTA AGT AAG CAC GGG TA | (Lee et al., 2023) |
| TcpH-SacIR | CCC GAG CTC GCT GCA GAT AAA TAA ATG CC |  |
| toxT-NcoⅠF | GGG CCA TGG CCA TTG GGA AAA AAT CTT TTC AAA | This study |
| toxT-SalⅠHisR | CCG GTC GAC TTA *GTG GTG GTG GTG GTG GTG* TTT TTC TGC AAC TCC TGT CAA CA |  |
| TcpJ-SalⅠF | GGG GTC GAC ATG GAA TAC GTT TAC TTG | This study |
| TcpJ-SalⅠR | GGG GTC GAC TCA CAT TAA ACG GAT TG |  |
| pBAD-toxT-XbaⅠF | GGC TCT AGA ATG GCC ATT GGG AAA | This study |
| pBAD-tcpJ-SacⅠR | CGG GAG CTC TCA CAT TAA ACG GAT TG |  |
| **Primers for RT-PCR** | | |
| *gyrA*-2F | AAT GTG CTG GGC AAC GAC TG | (Lo Scrudato and Blokesch, 2012) |
| *gyrA*-2R | GAG CCA AAG TTA CCT TGG CC |  |
| *toxT*-3 RT F | TGG GCA GAT ATT TGT GGT GA | (Abuaita and Withey, 2011) |
| *toxT*-3 RT R | AAA CGC TAG CAA ACC CAG AC |  |
| *tcpA* RT-F2 | ACC GGT CAA GAG GGT ATG AC | This study |
| *tcpA* RT-R2 | CAT ATT CTG CGA ATC AAT CGC AC |  |
| *ctxA*-2F | GGA GGG AAG AGC CGT GGA T | (Chtterjee et al., 2010) |
| *ctxA*-2R | CAT CGA TGA TCT TGG AGC ATT C |  |
