# Supplementary material for: Strain-dependent toxT expression, rather than ToxT activity, governs virulence gene regulation in Vibrio cholerae
Source: Front Microbiol. 2026 Feb 19;17:1755947. doi: 10.3389/fmicb.2026.1755947 (PMC12960492; doi:10.3389/fmicb.2026.1755947)
Supplement: Supplementary file 7 [file Table_3.docx]

**Table S3. CTXΦ Transduction efficiency of selected *V. cholerae* strains.**

|  | **O395**  (*toxT*-SY) | **O395**  (37°C) | **O395-H**  (*toxT*-SY-His) | **O395-H**  (37°C) | **EJK009**  (*toxT*-AF) | **EJK009**  (37°C) | **EJK009-H**  (*toxT*-AF-His) | **EJK009-H**  (37°C) |
| --- | --- | --- | --- | --- | --- | --- | --- | --- |
| No. of  transductants /  recipient cells | $\frac{\left( (387\pm17)\times{10}^{5} \right)}{3.0\times{10}^{8}}$ | $\frac{4 \pm2}{2.7\times{10}^{8}}$ | $\frac{(\left( 346\pm21)\times{10}^{5} \right)}{2.7\times{10}^{8}}$ | $\frac{4 \pm2}{2.6\times{10}^{8}}$ | $\frac{(\left( 931\pm37)\times{10}^{5} \right)}{3.5\times{10}^{8}}$ | $\frac{3 \pm2}{2.6\times{10}^{8}}$ | $\frac{(\left( 784\pm54)\times{10}^{5} \right)}{2.9\times{10}^{8}}$ | $\frac{\left( 3\pm2 \right)}{2.8\times{10}^{8}}$ |
| Transduction  Efficiency (%) | 12.9 ± 0.6 | <10^-5^ | 12.8 ± 0.8 | <10^-5^ | 26.6 ± 1.1* | <10^-5^ | 27.0 ± 1.9* | <10^-5^ |

Data represent the mean ± standard deviation from three independent experiments.

Transduction efficiencies at 30°C were compared between the *toxT*-SY group (O395/O395-H) and the *toxT*-AF group (EJK009/EJK009-H) using an unpaired two-tailed t-test (**p* < 0.05).
