# Supplementary material for: Strain-dependent toxT expression, rather than ToxT activity, governs virulence gene regulation in Vibrio cholerae
Source: Front Microbiol. 2026 Feb 19;17:1755947. doi: 10.3389/fmicb.2026.1755947 (PMC12960492; doi:10.3389/fmicb.2026.1755947)
Supplement: Supplementary file 8 [file Table_4.docx]

**Table S4. *tcpA* expression in *V. cholerae* strains carrying alternative *toxT* alleles under different culture conditions.”**

| **Cultured in LB medium at 30**°C | | | | | | | | |
| --- | --- | --- | --- | --- | --- | --- | --- | --- |
| O395 derivatives | *toxT* allele | *tcpA* expression | N16961 derivatives | *toxT* allele | *tcpA* expression | IB5230 derivatives | *toxT* allele | *tcpA* expression |
| O395 | *toxT*-SY | ○ | N16961 | *toxT*-SY | × | IB5230 | *toxT*-SY | × |
| O395-H | *toxT*-SY-His | ○ | N16961-H | *toxT*-SY-His | × | IB5230-H | *toxT*-SY-His | × |
| YJB001 | *toxT*-SF | ○ | YJB003 | *toxT*-SF | × | YJB020 | *toxT*-SF | ○ |
| YJB001-H | *toxT*-SF-His | ○ | YJB003-H | *toxT*-SF-His | × | YJB020-H | *toxT*-SF-His | ○ |
| EJK008 | *toxT*-AY | ○ | DHL008 | *toxT*-AY | ○ | DHL020 | *toxT*-AY | × |
| EJK008-H | *toxT*-AY-His | ○ | DHL008-H | *toxT*-AY-His | ○ | DHL020-H | *toxT*-AY-His | × |
| EJK009 | *toxT*-AF | ○ | DHL009 | *toxT*-AF | ○ | DHL021 | *toxT*-AF | × |
| EJK009-H | *toxT*-AF-His | ○ | DHL009-H | *toxT*-AF-His | ○ | DHL021-H | *toxT*-AF-His | × |
| **Cultured in LB medium at 37**°C | | | | | | | | |
| O395 derivatives | *toxT* allele | *tcpA* expression | N16961 derivatives | *toxT* allele | *tcpA* expression | IB5230 derivatives | *toxT* allele | *tcpA* expression |
| O395 | *toxT*-SY | × | N16961 | *toxT*-SY | × | IB5230 | *toxT*-SY | ○ |
| O395-H | *toxT*-SY-His | × | N16961-H | *toxT*-SY-His | × | IB5230-H | *toxT*-SY-His | ○ |
| YJB001 | *toxT*-SF | × | YJB003 | *toxT*-SF | × | YJB020 | *toxT*-SF | ○ |
| YJB001-H | *toxT*-SF-His | × | YJB003-H | *toxT*-SF-His | × | YJB020-H | *toxT*-SF-His | ○ |
| EJK008 | *toxT*-AY | × | DHL008 | *toxT*-AY | × | DHL020 | *toxT*-AY | × |
| EJK008-H | *toxT*-AY-His | × | DHL008-H | *toxT*-AY-His | × | DHL020-H | *toxT*-AY-His | × |
| EJK009 | *toxT*-AF | × | DHL009 | *toxT*-AF | × | DHL021 | *toxT*-AF | × |
| EJK009-H | *toxT*-AF-His | × | DHL009-H | *toxT*-AF-His | × | DHL021-H | *toxT*-AF-His | × |
